# A new perspective: deciphering the aberrance and clinical implication of disulfidptosis signatures in clear cell renal cell carcinoma

**DOI:** 10.18632/aging.205916

**Published:** 2024-06-10

**Authors:** Bohong Chen, Mingguo Zhou, Li Guo, Xinyue Sun, Haoxiang Huang, Kaijie Wu, Wei Chen, Dapeng Wu

**Affiliations:** 1 Department of Urology, The First Affiliated Hospital of Xi’an Jiaotong University, Xi’an 710061, Shaanxi, China; 2 Department of neurology, The First Affiliated Hospital of Xi’an Jiaotong University, Xi’an 710061, Shaanxi, China

**Keywords:** disulfidptosis, pan-cancer, clear cell renal cell carcinoma, molecular subtypes, single-cell RNA-seq

## Abstract

Recent research has discovered disulfidptosis as a form of programmed cell death characterized by disulfide stress. However, its significance in clear cell renal cell carcinoma (ccRCC) remains unclear. To investigate this, data from The Cancer Genome Atlas were collected and used to identify ccRCC subgroups. Unsupervised clustering was employed to determine ccRCC heterogeneity. The mutation landscape and immune microenvironment of the subgroups were analyzed. The Disulfidptosis-Related Score was calculated using the LASSO-penalized Cox regression algorithm. The E-MATB-1980 cohort was used to validate the signature. The role of SLC7A11 in ccRCC metastasis was explored using western blotting and Transwell assays. Disulfidptosis-related genes are commonly downregulated in cancers and are linked to hypermethylation and copy number variation. The study revealed that ccRCC is divided into two sub-clusters: the disulfidptosis-desert sub-cluster, which is associated with a poor prognosis, a higher mutation frequency, and an immunosuppressive microenvironment. A 14-gene prognostic model was developed using differentially expressed genes and was validated in the E-MATB-1980 cohort. The low-risk group demonstrated longer overall and disease-free survival and responded better to targeted immunotherapy. Results from *in vitro* experiments identified SLC7A11 as a key participant in ccRCC metastasis.

## INTRODUCTION

Cancer is a leading cause of death worldwide, accounting for approximately 65% of all deaths [1]. As the human lifespan increases, the incidence and mortality of cancer also increase. Renal cell carcinoma (RCC) is a common malignancy of the genitourinary system. Clear cell RCC (ccRCC) accounts for approximately 75% of all RCC cases and is an aggressive subtype [2]. At the time of diagnosis, 20-30% of patients already have metastases. Even after surgical removal of the tumor, nearly 30% of the patients experience recurrence and metastasis [3, 4]. Furthermore, advanced metastatic RCC shows a limited response to radiotherapy and chemotherapy, resulting in a restricted range of clinical treatment options and poor prognosis [5–7]. The 5-year survival rate of patients with metastatic renal cancer is less than 5% [8]. The TNM staging system is commonly used to predict kidney cancer prognosis. However, its predictive ability is limited because the survival rate can vary significantly among patients with the same stage of the disease [9]. Several prognostic models are available for predicting the outcomes of ccRCC. Bian et al. developed a predictive model for recurrence-free survival (RFS) in patients using conventional clinical variables, such as prothrombin time, albumin/globulin ratio, platelet count, sex, and fibrinogen levels [10]. Meng et al. investigated the prognostic value of cell division cycle-related proteins [11] in accurately predicting RFS. However, no study has explored the role of disulfide-related genes in ccRCC prognosis.

Disulfidptosis is a recently discovered form of cell death that is distinct from cuprotosis, apoptosis, ferroptosis, pyroptosis, and necroptosis [12]. It is characterized by abnormal buildup of intracellular disulfides, particularly cystine, which causes disulfide stress and exerts significant toxicity on cells [13, 14]. In cancer cells with abnormal SLC7A11 expression, high rates of cystine uptake and reduction to cysteine, combined with glucose starvation, deplete the NADPH pool. This depletion results in a massive accumulation of intracellular disulfide molecules. Anomalous accumulation of intracellular disulfides leads to aberrant disulfide bonds in actin cytoskeleton proteins and the collapse of F-actin in an SLC7A11-dependent manner, ultimately resulting in rapid cell death. Alterations in the cytoskeleton of plants and animals play an active role in initiating and regulating PCD [15, 16]. Inhibition of glucose transporters to induce disulfidoptosis may be an effective therapeutic strategy for treating SLC7A11 high tumors, which frequently occur in human cancers [12, 17]. Therefore, investigating the mechanism of disulfidoptosis in tumors is important to induce cancer cell death and tumor killing.

This study characterized disulfidptosis-related genes using pan-cancer analysis and stratified patients with ccRCC by integrating multiomics data. The study included prognostic, enrichment, gene mutation, immune infiltration, and single-cell analyses. A reliable risk stratification model was constructed to predict the prognosis and therapeutic response of patients with ccRCC. SLC7A11 is closely associated with metastasis and may serve as a therapeutic target in ccRCC. It is an important prognostic model component gene.

## RESULTS

### Dysregulation and mutation of disulfidptosis-related genes in cancers

**Figure 1:**
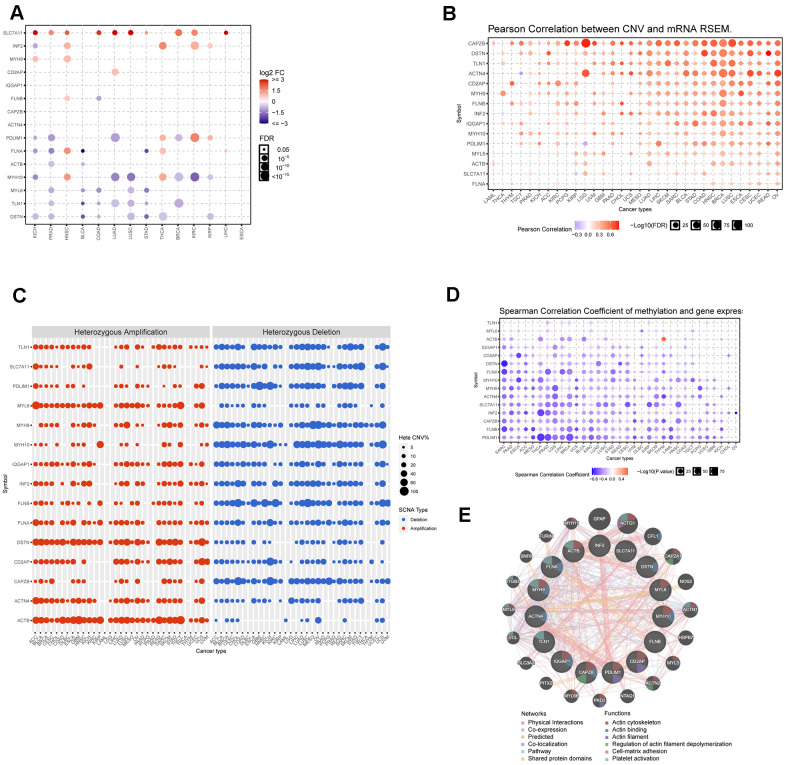
**Dysregulation, mutation, and methylation of disulfidptosis-related genes in cancers.** (**A**) Expression of disulfidation-related genes in multiple cancerous and normal tissues. (**B**) Bubble chart showing the correlation between CNV and the expression of disulfidptosis-related genes. (**C**) Heterozygous and homozygous amplification/deletion of disulfide-related genes in multiple cancers. (**D**) Bubble chart showing the correlation between methylation of the 15 disulfide-related molecules and mRNA expression. (**E**) PPI network of 15 disulfide-related genes.

Disulfidptosis has become an increasingly important focus of cancer research. Initially, we examined the expression patterns of disulfidptosis-related genes in various cancer types. As shown in Figure 1A, most disulfidoptosis genes were downregulated in multiple cancer types, including PDLIM1, TLN1, and MYH10 in lung squamous cell carcinoma (LUSC), MYL6 and DSTN in prostate adenocarcinoma (PRAD), and FLNA and ACTB in bladder cancer (BLCA). These results indicated that disulfidptosis is suppressed in cancer cells. Therefore, drugs that can induce disulfidoptosis in cancer cells may enhance their therapeutic efficacy in tumor treatment. Furthermore, SLC7A11, IFN2, and MYH9 were significantly upregulated in kidney clear cell carcinoma (KIRC) and head and neck squamous cell carcinoma (HNSC). To gain a comprehensive understanding of the dysregulated expression of disulfidoptosis genes, we investigated copy number variation (CNV) and single-nucleotide variations (SNV) across various cancers (Figure 1B, 1C). Our findings indicated a significant correlation between CNV and gene expression in most cancers, particularly BRCA, COAD, LUSC, and OV. As shown in Figure 1C, heterozygous amplifications frequently appeared in ACTB, DSTN, and MYL6, whereas heterozygous deletions often occurred in CAPZB, FLNB, MYH10, and PDL1M1. The frequency of SNVs in genes associated with disulfidoptosis was analyzed. 94.76 Of the tested genes, 94.76% had at least one mutation site (Additional File 1, Supplementary Figure 2A). FLNA, FLNB, MYH9, and TLN1 displayed high mutation frequencies, with FLNA having the highest SNV rate (25%). Cancers with high SNV rates included UCEC, SKCM, STAD, and COAD (Supplementary Figure 2B). Methylation was found to have a negative correlation with gene expression. The expression of most disulfidoptosis genes, such as DSTN, FLNA, INF2, and PDL1M1, was negatively correlated with the methylation status (Figure 1D). This suggests that most disulfidoptosis genes are hypermethylated in various cancers. The hypermethylated genes SLC7A11 and CD2AP were primarily associated with better prognosis in cancer, whereas the hypomethylated gene MYL6 was predominantly associated with poor prognosis in PCPG (Supplementary Figure 2C). Moreover, we conducted a Protein-Protein Interaction (PPI) analysis using the GeneMANIA website and found that MYH9, ACTB, and PDLIM1 were hub genes in the interaction network (Figure 1E).

### Establishment of two clusters by clustering analysis of disulfidptosis genes in ccRCC

**Figure 2:**
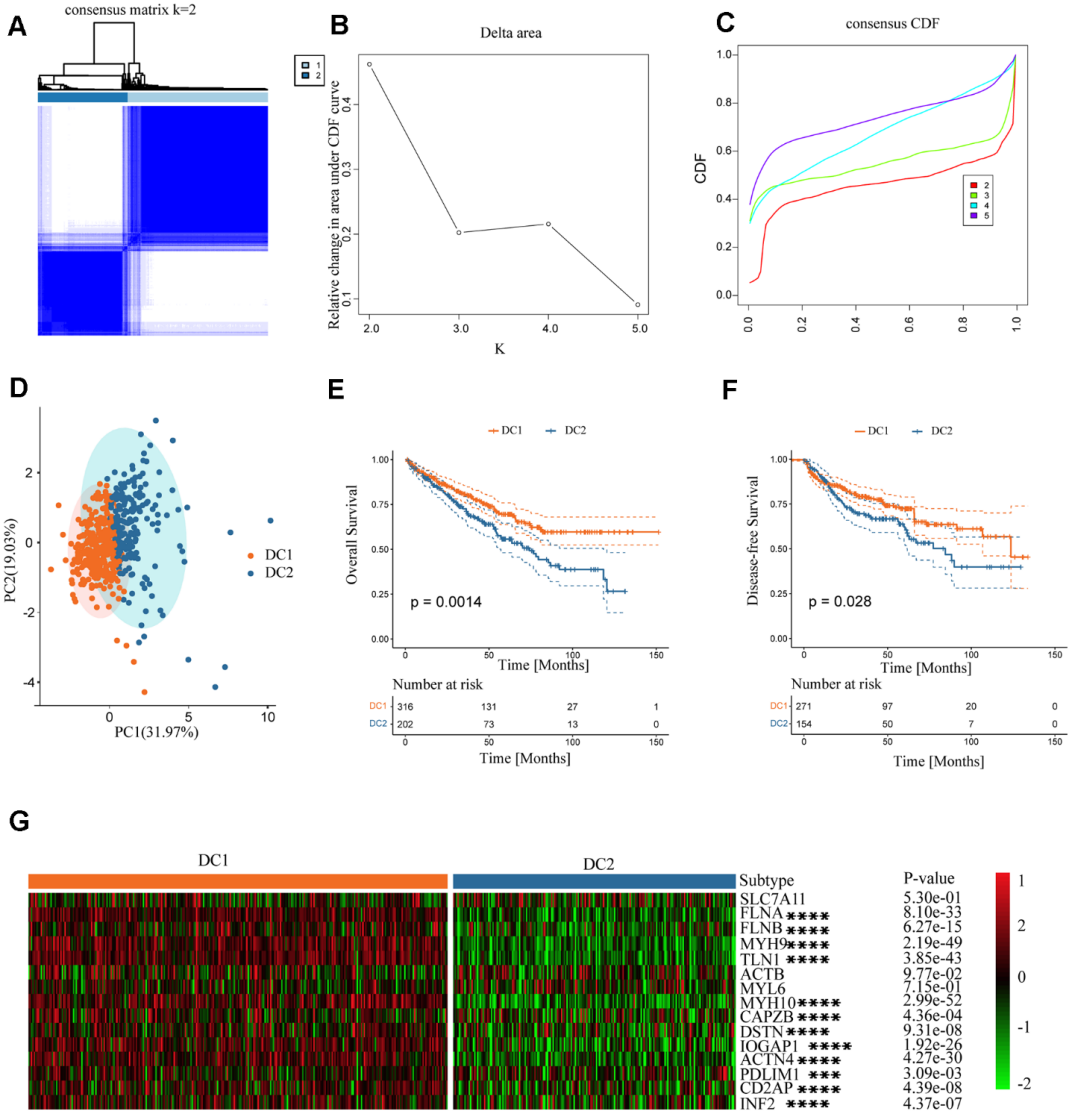
**Establishment of two clusters of disulfide-related genes in ccRCC.** (**A**) Consensus matrix of the samples in TCGA-KIRC for k = 2. (**B**) Cluster numbers are determined by the lowest proportion of ambiguous clusters. (**C**) Cumulative distribution function curves, k = 2–5. (**D**) Principal component plot based on disulfide-related genes. (**E**, **F**) Survival analysis of overall survival (left) and disease-free survival (right) of the two subtypes in the TCGA-KIRC dataset. (**G**) Expression profiles of disulfide-related genes in the two subtypes.

There was a notable difference in the expression of the disulfidoptosis genes between ccRCC and normal tissues. However, the implications of these signatures in cancers, particularly ccRCC, are not yet fully understood. Therefore, we investigated the characteristics of disulfide pathogenesis-related genes in ccRCC. An unsupervised clustering method was used to classify TCGA ccRCC samples into distinct subtypes based on the expression levels of the disulfidoptosis-related genes. The optimal number of clusters (K) for the analysis was determined using the K value derived from the cumulative distribution function (CDF) with a minimal slope of decline, ensuring the highest consistency. The TCGA-KIRC dataset was optimally categorized into two subtypes, disulfidoptosis-cluster 1 (DC1) and cluster 2 (DC2), with k=2 determined to be the best classification number (Figure 2A–2D). Patients with the DC2 subtype exhibited significantly shorter overall survival (OS) and disease-free survival (DFS) than those with the DC1 subtype (Figure 2E, 2F). Furthermore, we analyzed the expression levels of disulfidoptosis-related genes in the ccRCC subtypes. The DC2 subtype, characterized by the depletion of disulfidoptosis-related genes, showed lower expression levels of these genes than the DC1 subtype (Figure 2G). Downregulation of disulfidoptosis-related genes in the DC2 subtype contributes to the suppression of disulfidoptosis, making it a prognostic factor associated with poor outcomes.

### Functional enrichment analysis of ccRCC subtypes

**Figure 3:**
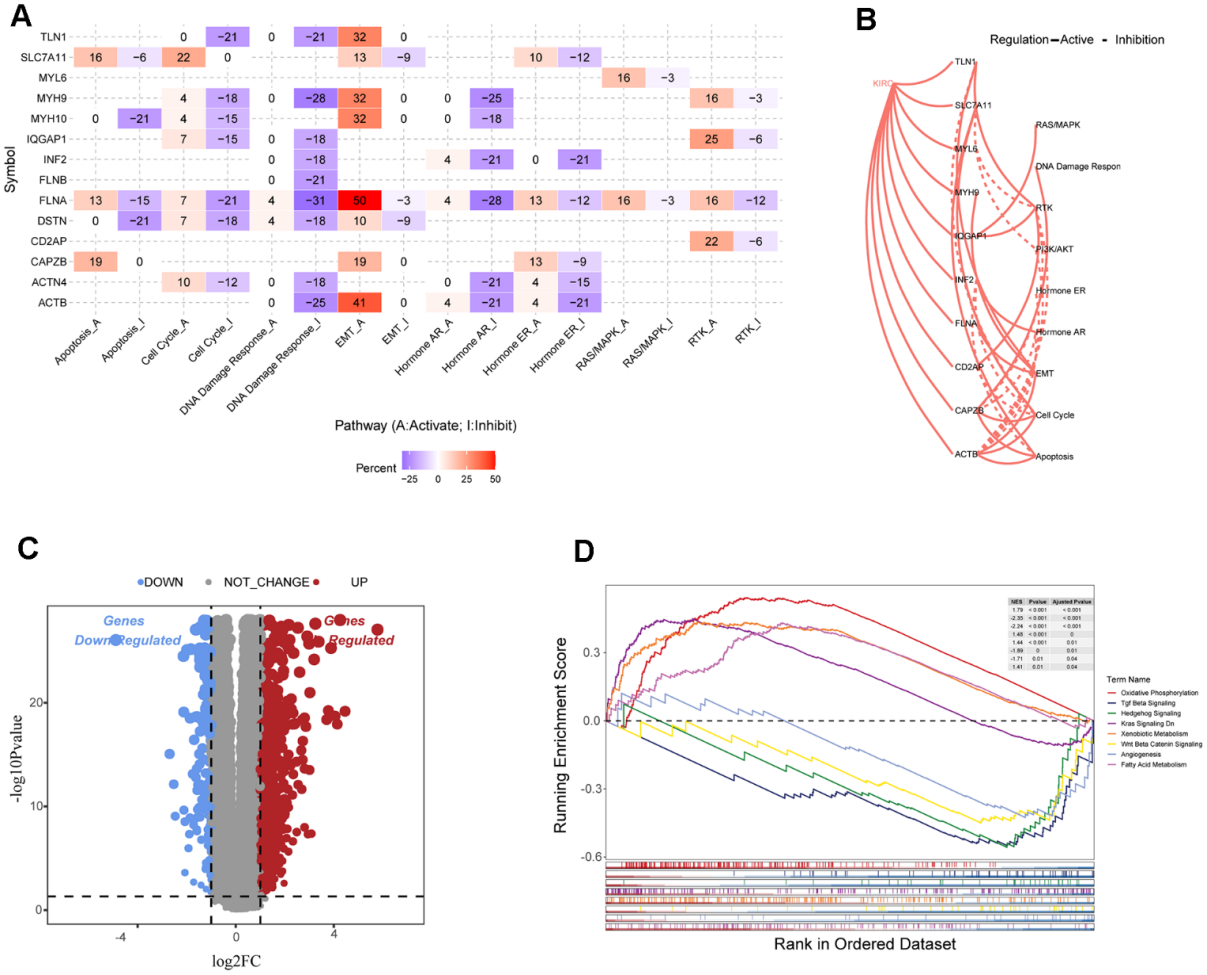
**Analysis of disulfide-related signaling pathways.** (**A**) Heatmap showing the correlation between the expression levels of 15 disulfidoptosis-related molecules in important cancer signaling pathways. (**B**) Correlation between 15 disulfidation-related molecules in ccRCC and important cancer signaling pathways. The solid line represents activation and the dashed line represents inhibition. (**C**) Volcano plot of DEGs between the two clusters. (**D**) GSEA enrichment analysis of specific biological pathways in the two disulfidptosis phenotypes.

This study analyzed differences in gene expression among disulfidoptosis subtypes and investigated the associated signaling pathways. Most signaling pathways in pan-cancer, including ACTB, FLNA, MYH9, MYH10, and TLN1, exhibited high activation levels in the epithelial-mesenchymal transition (EMT) pathway, but consistent inhibition of the cell cycle, DNA damage response, and hormone AR (Figure 3A). In renal cancer tissue, most disulfidoptosis molecules activated apoptosis, EMT, cell cycle, and RTK signaling pathways, but inhibited the PI3K/AKT, hormone ER, and hormone AR signaling pathways (Figure 3B). To further investigate the molecular differences between the DC1 and DC2 subtypes and their potential implications, we examined the differentially expressed genes (DEGs) between these subgroups using the TCGA database (Figure 3C). We performed gene set enrichment analysis (GSEA) to identify enriched pathways associated with subtypes DC1 and DC2. The analysis revealed that the DC1 subtype was primarily enriched in pathways related to oxidative phosphorylation, KRAS signaling, xenobiotic metabolism, and fatty acid metabolism. In contrast, the DC2 subtype showed stronger correlations with pathways such as TGF Beta signaling, hedgehog signaling, Wnt β-catenin signaling, and angiogenesis (Figure 3D). These findings indicate that the two subtypes have different molecular characteristics and signaling pathways, which may contribute to their different prognoses in ccRCC.

### Comparison of tumor somatic mutations and CNVs in two subtypes

**Figure 4:**
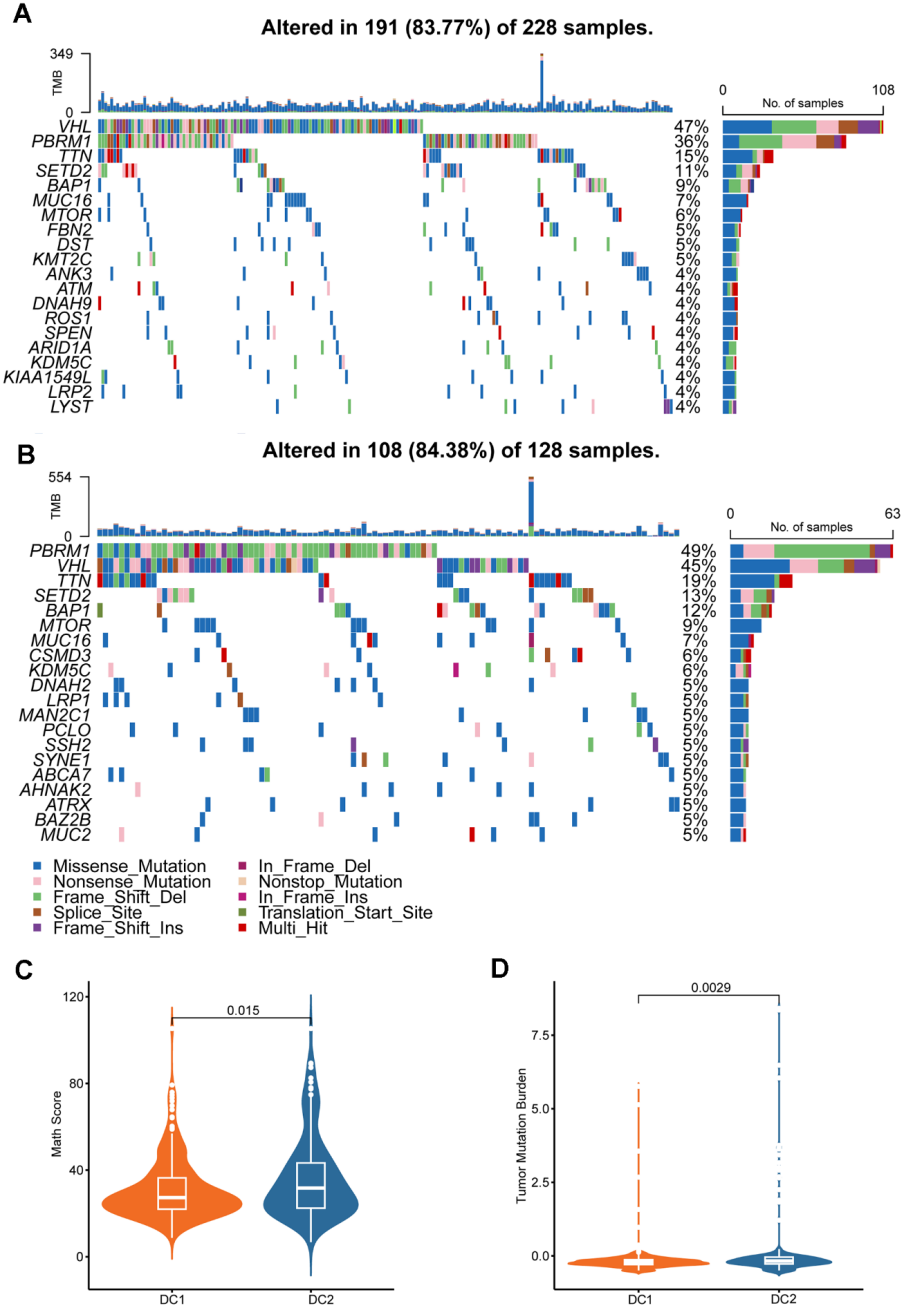
**Genomic alterations in disulfidptosis-related signatures.** Genomic profiling of the 20 most frequently altered genes in the DC1 (**A**) and DC2 (**B**) groups. (**C**) Comparison of MATH score between the two ccRCC subtypes. (**D**) Comparison of the TMB levels between the two ccRCC subtypes.

To explore the characteristics of the two RCC subtypes comprehensively, we investigated the differential distribution of tumor somatic mutations between them. Our analysis revealed that the DC2 subtype had a higher frequency of gene mutations than did the DC1 subtype (Figure 4A, 4B). The graph illustrates the mutation frequencies of the top 20 mutated genes. In both subtypes, the most frequently mutated genes were VHL, PBRM1, TTN, SETD2, and BAP1. The DC2 subtype exhibited higher mutation frequencies in several genes compared to the DC1 subtype, including VHL (58.5% vs. 57.9%), PBRM1 (55.3% vs. 46.1%), TTN (22.3% vs. 19.1%), BAP1 (13.8% vs. 11.2%), and MUC16 (9.6% vs. 4%). The difference in PBRM1 mutational frequency between the two subtypes was statistically significant (*p <* 0.05; Supplementary Table 1). The distribution of mutant allele tumor heterogeneity (MATH) scores between the two subtypes (Figure 4C) was consistent with this observation. Furthermore, differences in tumor mutation burden (TMB) were observed between the two groups (Figure 4D), suggesting an association between gene mutations and the ccRCC subtype phenotypes.

### Comparison of immune infiltration characteristics between subtypes

**Figure 5:**
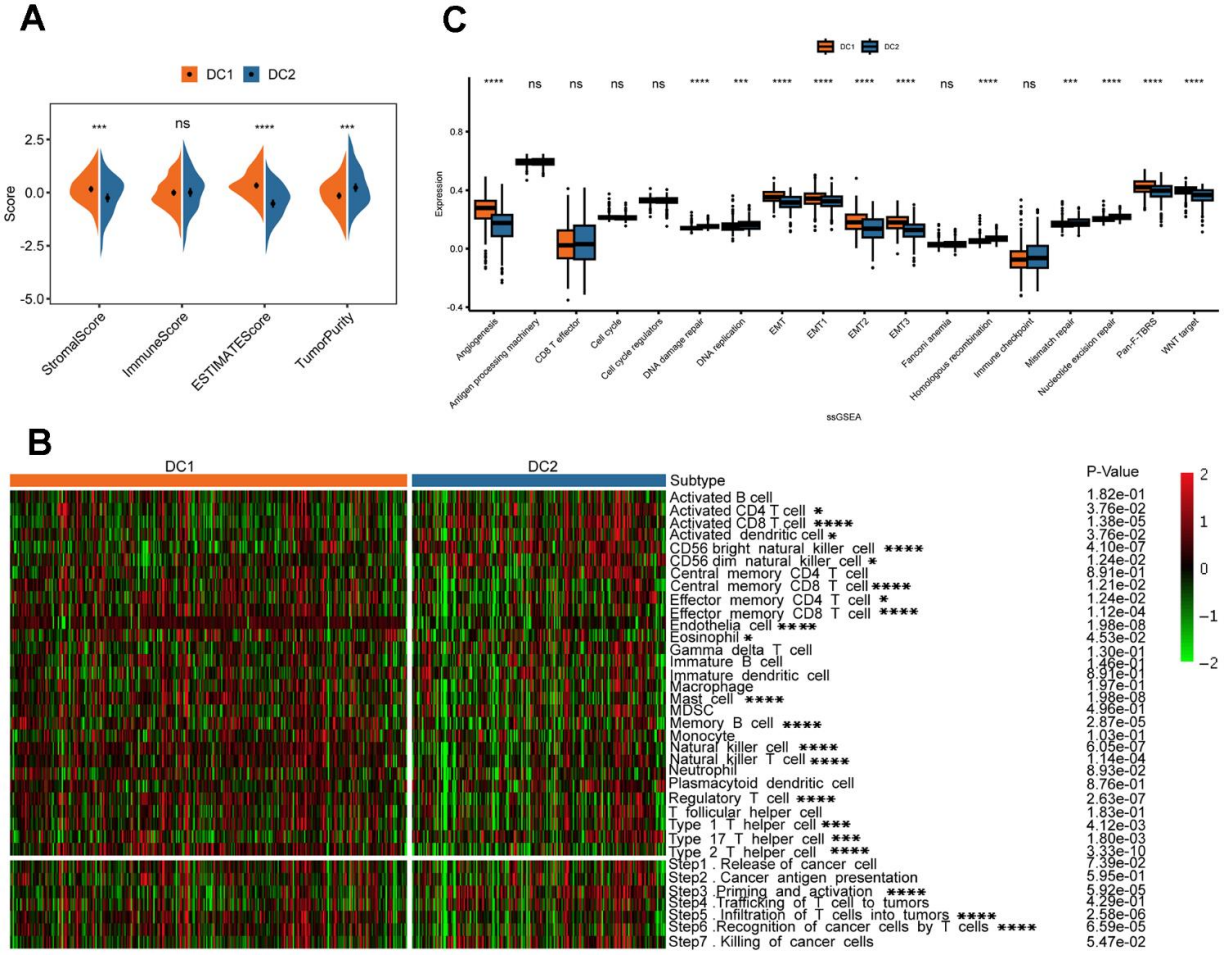
**Variations in TME in the two disulfidptosis phenotypes.** (**A**) The result of estimation between the two disulfidoptosis phenotypes from TCGA-KIRC datasets. ns, *p >* 0.05; ***p < 0.001, ****p < 0.0001. (**B**) The heatmap showing the frequency of TME-infiltrating cells and cancer immune cycle among the two disulfidoptosis phenotypes based on ssGSEA. (**C**) The Wilcox test was used to evaluate the TME-related scores of different disulfidoptosis patterns. ns, *p >* 0.05; ***p < 0.001, ****p < 0.0001.

The effectiveness of drug therapy is influenced not only by genomic mutations but also by the tumor immune microenvironment. Therefore, we examined the relationship between disulfidoptosis subtypes and the tumor microenvironment in a cohort of 518 patients from TCGA using the ESTIMATE and ssGSEA algorithms. Analysis using ESTIMATE revealed that the DC2 group had a higher tumor purity than the DC1 group (Figure 5A). Furthermore, there was an inverse relationship between the stromal and ESTIMATE scores. Although the difference was not statistically significant, the immune score decreased in the DC2 group. Moreover, the ssGSEA analysis revealed a significant decrease in the proportion of various immune cell types that have antitumorigenic properties in the DC2 group. These include adaptive immune cells, such as central memory CD8 T cells, effector memory CD4 T cells, effector memory CD8 T cells, memory B cells, regulatory T cells, type 1 T helper cells, and type 2 T helper cells, as well as innate immune cells, such as endothelial cells, eosinophils, mast cells, natural killer cells, natural killer T cells, and neutrophils (Figure 5B). In contrast, the DC2 group exhibited significant enrichment of activated B cells, activated CD4 T cells, activated CD8 T cells, activated dendritic cells, and type 17 T helper cells. We subsequently analyzed the tumor immune cycle-related signals among the subtypes of disulfidoptosis. The antitumor immune response requires successful completion of a series of sequential events collectively known as the cancer immune cycle. The results of this study suggest that the DC1 subtype has active pathways in steps 3, 5, and 6 (Figure 5B, bottom), supporting the significant role of disulfidoptosis in cancer infiltration. In addition, we analyzed immune-related gene set scores and found that both Pan-F-TBRS and Wnt target gene sets had higher values for the DC1 subtype (Figure 5C). These findings indicate that tumors in the DC2 group may create an immunosuppressive microenvironment, which hinders antitumor immunity, promotes tumor growth, and leads to a poorer prognosis.

### Construction of the DRS for patients with ccRCC

**Figure 6:**
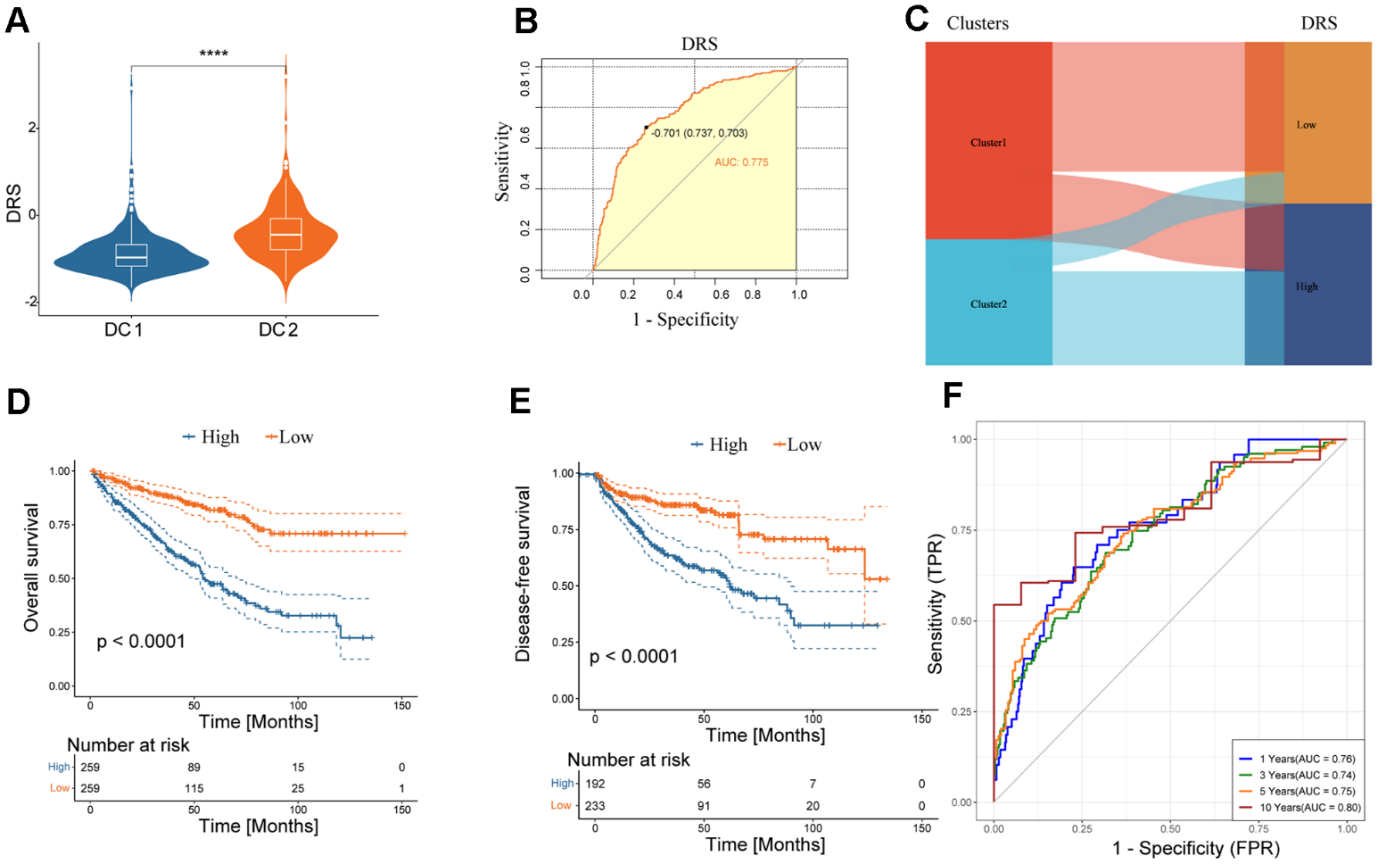
**Clinical significance of DRS.** (**A**) Boxplots show DRS at two levels of disulfidptosis. ****p < 0.0001. (**B**) ROC curve analysis of the predictive value of DRS for disulfidptosis phenotypes. (**C**) Alluvial plot showing the association between DRS and disulfidoptosis phenotypes. (**D**, **E**) Survival analysis of overall survival (left) and disease-free survival (right) of the two DRS groups in the TCGA-KIRC dataset. (**F**) Time-dependent ROC analysis of the predictive value of DRS for the overall survival of patients at 1, 3, 5, and 10 years.

The study confirmed the significance of disulfidptosis in regulating the tumor immune microenvironment and predicting survival in patients with renal cancer. A scoring model, known as the disulfidation-related score (DRS), was developed based on the DEGs between the DC1 and DC2 subtypes. Initially, a univariate Cox regression analysis of the 1102 DEGs was conducted. Subsequently, 278 genes with a *p* < 0.01 were included for further multivariate Cox regression analysis. A DRS was constructed using a LASSO regression analysis of 33 selected molecules from multivariate Cox regression. The coefficients obtained and the expression levels of each variable were used to construct the DRS, as shown in Supplementary Figure 3. A comparison of DRS levels between the two subtypes revealed higher DRS values in DC2, as shown in Figure 6A. The DRS accurately predicted the renal cancer subtypes, as shown in Figure 6B, 6C, with an area under the ROC curves (AUC) of 0.775. The high-DRS group exhibited worse OS and DFS than the low-risk group in both cohorts, as shown in Figure 6D, 6E and Supplementary Figure 4A. The DRS model’s high sensitivity and specificity in predicting prognosis was further confirmed by the area under the Receiver operating characteristic (ROC) curves. The AUC scores were 0.76, 0.74, 0.75, and 0.80 at 1, 3, 5, and 10 years, respectively, in the TCGA ccRCC cohort (Figure 6F). The prognostic model was validated in an external validation set, where the AUC values of the ROC curve at 3, 5, and 10 years were all above 0.7 (Supplementary Figure 4B), demonstrating the potential wide application of this prognostic model.

### Comparison of cell death-related prognostic signatures in ccRCC

**Figure 7:**
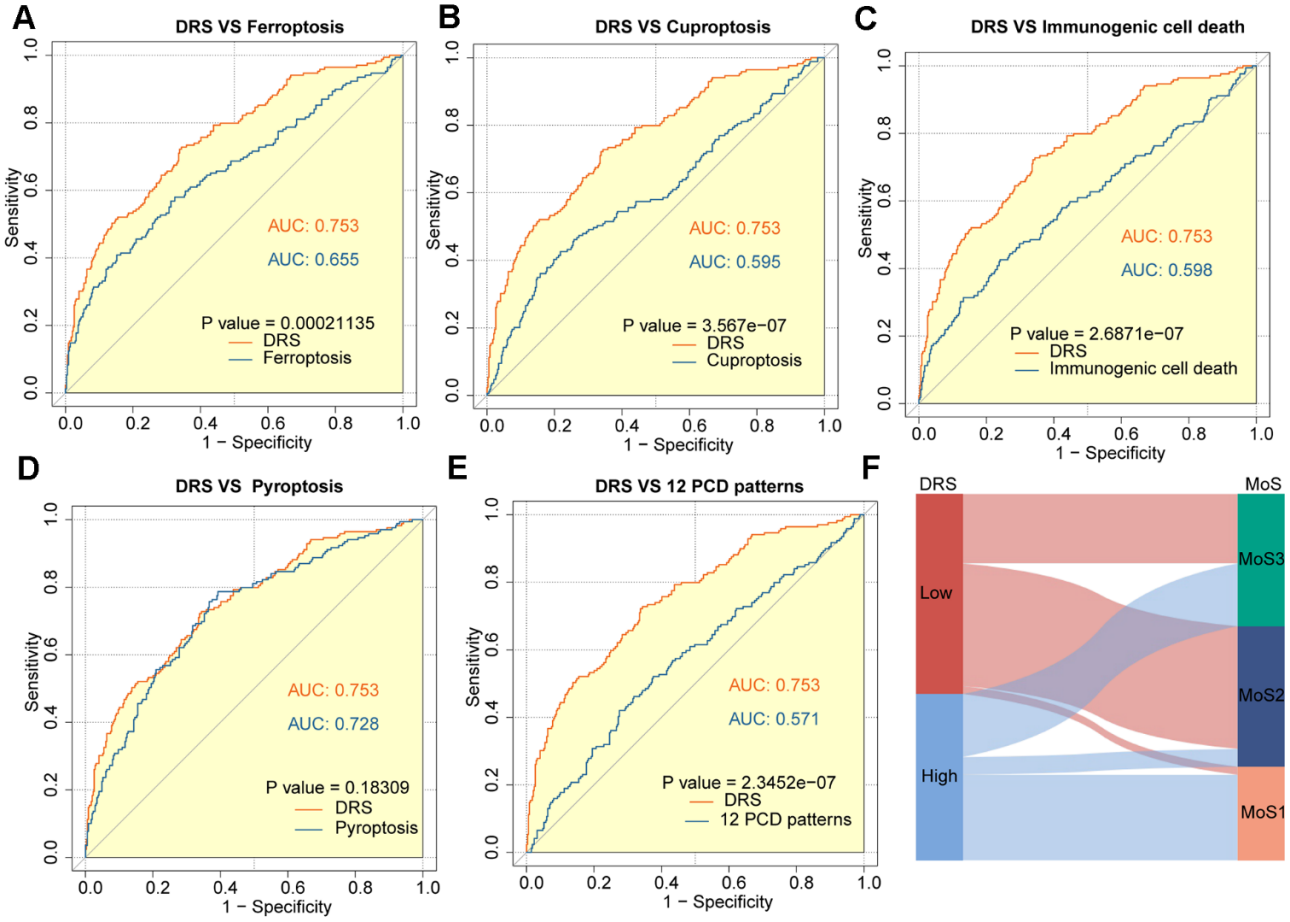
**Outstanding performance of DRS compared to other cell death-related models.** (**A**–**E**) AUC analysis of the DRS and other PCD-related models in the TCGA-KIRC cohort. (**F**) The alluvial plot shows the association between DRS and MoS subtypes.

In recent years, many prognostic signatures based on gene expression have been reported due to the emergence of next-generation sequencing technologies. We evaluated the prognostic accuracy of the DRS compared with five other cell death-related signatures: ferroptosis, cuproptosis, immunogenic cell death, pyroptosis, and a composite of 12 combined PCD models. DRS outperformed the other five risk scores in predicting patient survival, as indicated by the higher AUCs. This difference in performance was significant for all four signatures (*p* < 0.05, Figure 7A–7E). Furthermore, we observed that patients in the high-DRS group were more concentrated in the MoS1 category, which confers a worse prognosis, which is consistent with our findings. Conversely, patients with a low DRS were concentrated in the MoS2 category, which is associated with a more favorable prognosis. This finding was consistent with our results (Figure 7F).

### Nomogram establishment based on the multivariable Cox regression model

**Figure 8:**
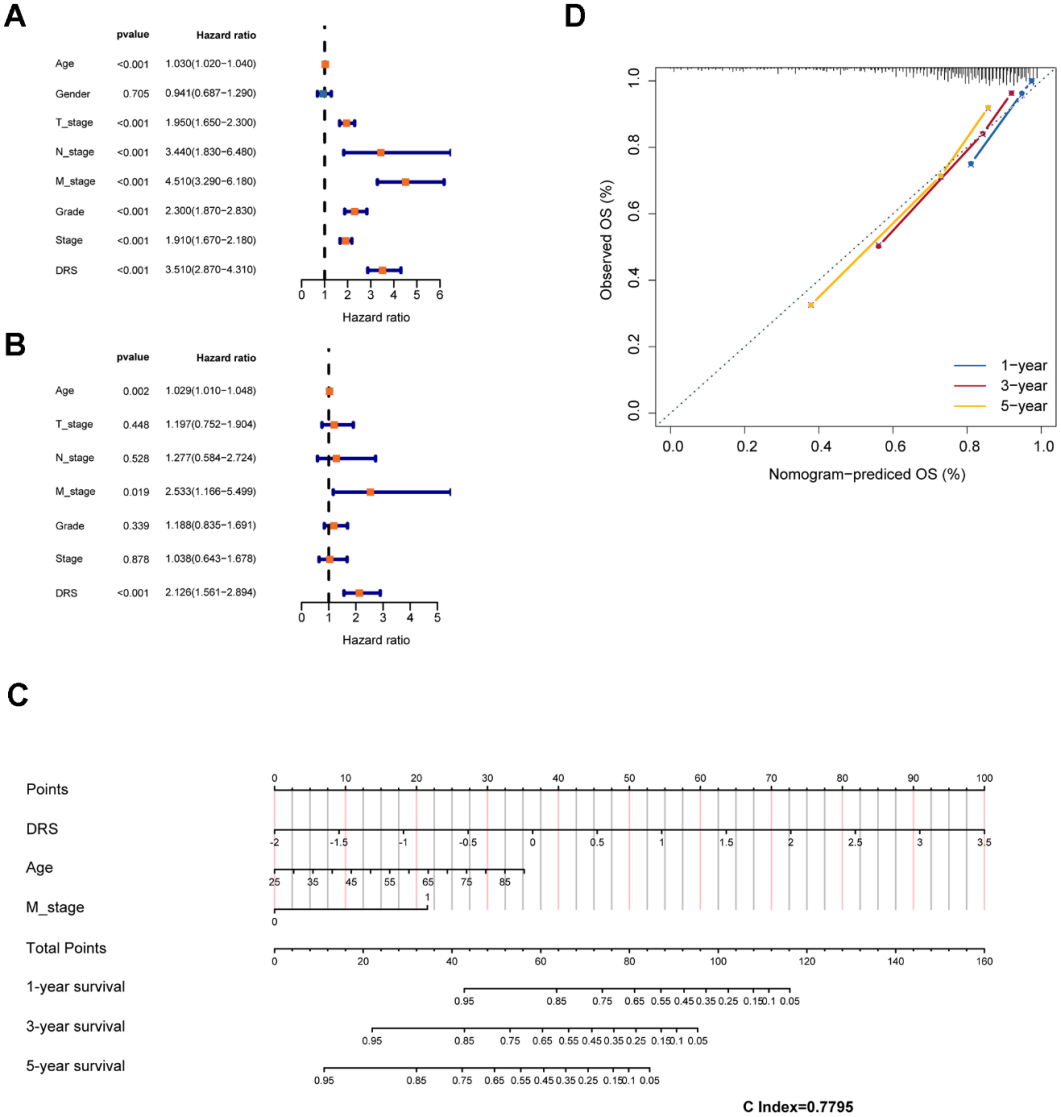
**Construction of a prognostic nomogram based on clinical features and DRS.** (**A**, **B**) Analysis of DRS and other clinical characteristics of TCGA-KIRC patients based on univariate and multivariate Cox models. (**C**) Use of DRS and other clinical features of patients with ccRCC to construct a prognostic nomogram for the training cohort. (**D**) Calibration curve analysis validating the stability of the model (1 year: blue; 3 years: red; 5 years: green).

Clinicopathological features, including age, sex, pathological TNM stage, pathological stage, and grade, were incorporated as independent prognostic factors in the univariate Cox analysis. The results showed that age, pathological TNM stage, pathological stage, grade, and DRS all had a *p <* 0.05, indicating their significant prognostic value (Figure 8A). These factors were included in the multivariate Cox model based on the findings of the univariate analysis (Figure 8B). Nomograms and calibration curves were constructed for the 1-, 3-, and 5-year predictions based on the independent prognostic factors identified using the multivariable Cox model (Figure 8C, 8D). The final predictors in the nomograms were DRS, pathologic M, and age, and the C-index was 0.7795, indicating the good discrimination ability of the nomogram prediction model.

### Disulfidptosis-related score was a potential diagnostic biomarker for ccRCC

**Figure 9:**
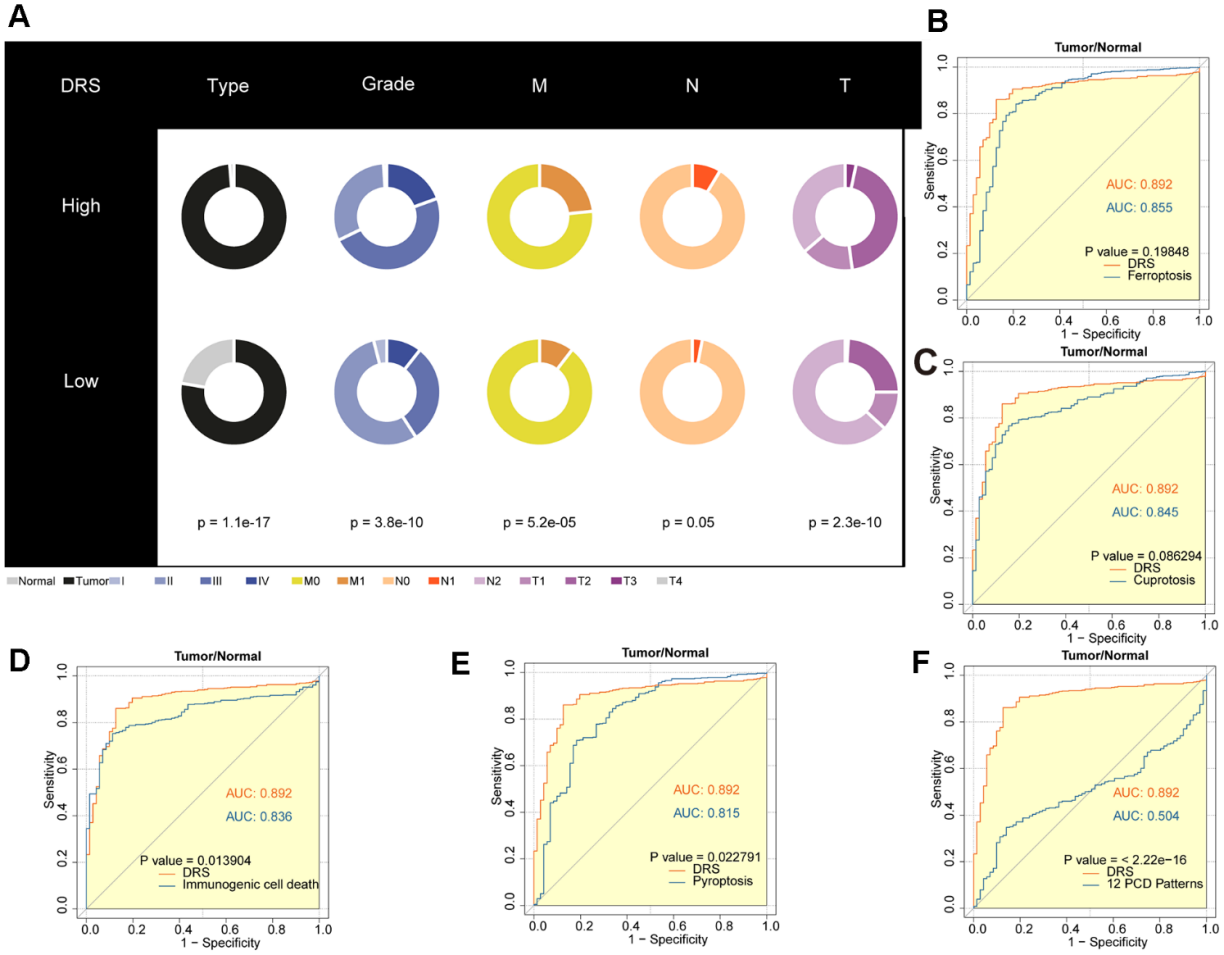
**Diagnostic value analysis of the DRS in ccRCC.** (**A**) Pie charts showing the chi-square test of clinicopathological factors for DRS in ccRCC. (**B**–**F**) AUC analysis of the DRS and other PCD-related models for distinguishing ccRCC from normal RCC. *p <* 0.05 was considered statistically significant.

To investigate the clinical utility of DRS, we compared the clinicopathological features of patients in the high- and low-DRS groups from the TCGA cohort. Chi-square test revealed that the high-DRS group had poorer tissue type, grade, and TNM staging than the low-DRS group, indicating an association between high DRS and more malignant ccRCC (Figure 9A). To improve the accuracy of ccRCC diagnosis, we evaluated the potential of the DRS as a specific and sensitive biomarker for early detection. We conducted ROC analysis to evaluate the diagnostic performance of DRS. As shown in Figure 9B–9F, the DRS outperformed the other five models in discriminating ccRCC from normal tissue samples. Statistical analysis revealed that the differences between DRS and the three comparative models were significant (*p <* 0.05, DeLong’s test), indicating the superior diagnostic capability of the proposed signature.

### Drug sensitivity analysis and DRS

**Figure 10:**
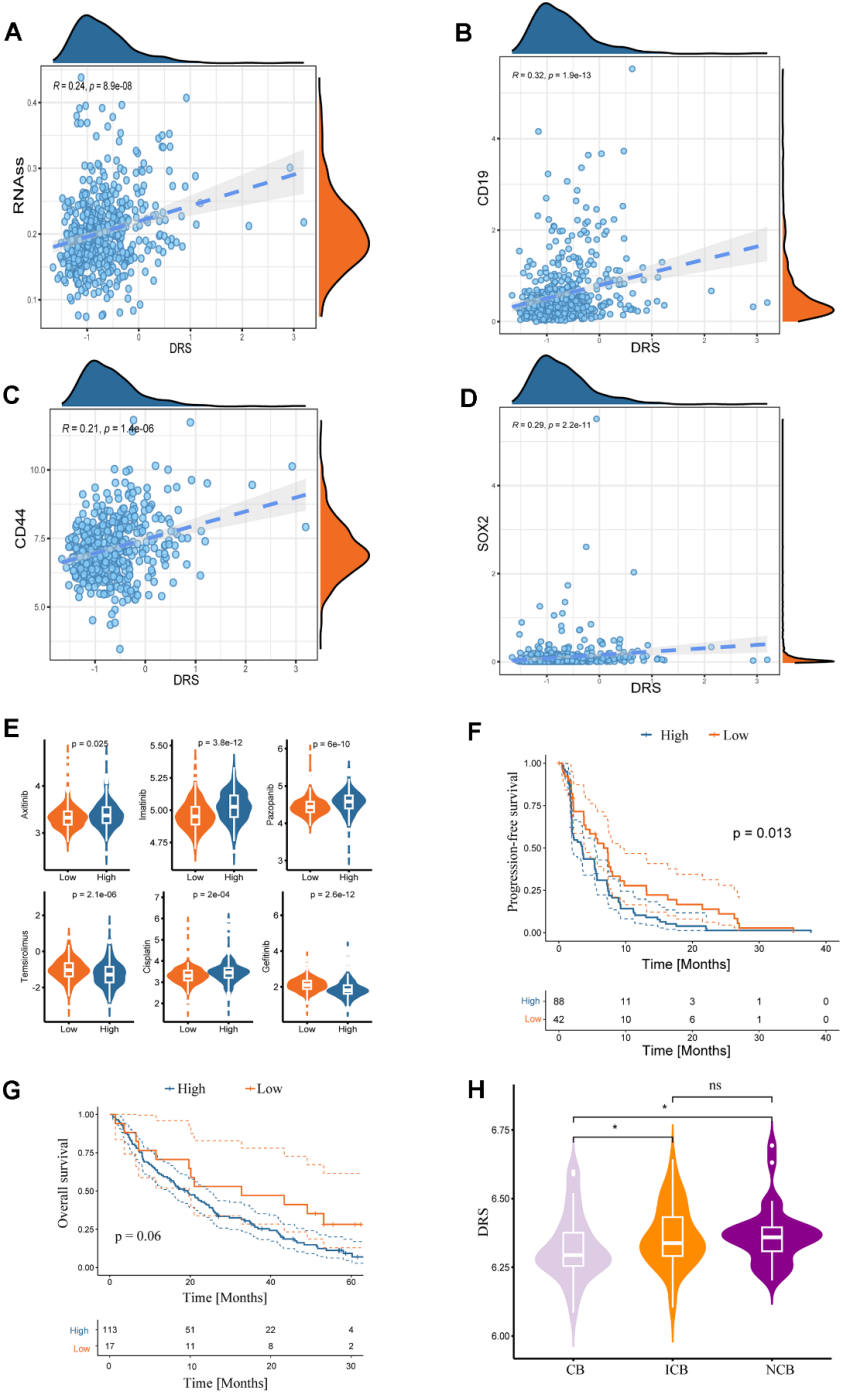
**Associations between DRS and stemness index and clinical drug treatment responses.** Correlation analysis of DRS with RNA stem score (**A**) and renal cancer stem cell markers (CD19, CD44, and SOX2) (**B**–**D**). Statistical significance was set at *p <* 0.05. (**E**) Boxplots of the estimated IC_50_ values of chemotherapeutic and targeted agents in the high- and low-DRS groups, including axitinib, imatinib, pazopanib, temsirolimus, cisplatin, and gefitinib. (**F**, **G**) Survival analysis for progression-free survival and overall survival of the two DRS groups in patients with ccRCC who were treated with everolimus from the RCC-Braun_2020 cohort. (**H**) Comparison of DRS of different patients with ccRCC in different remission states after targeted therapy in RCC-Braun_2020 cohort. (ns, *p >* 0.05; *p < 0.05).

A subset of tumor cells, known as tumor-initiating cells (TIC) or cancer stem cells (CSC), possesses stem cell-like properties that enable them to evade immune surveillance and exhibit resistance to current therapeutic interventions [18]. The study also measured the association between DRS and the RNA stem score (RNAss) [19]. Scatter plots and regression analyses revealed a significant positive correlation between the DRS and RNAss (Spearman correlation coefficient R = 0.24; *p =* 8.9e^-08^) (Figure 10A). Furthermore, DRS was strongly associated with cancer stem cell markers [20], including CD19 (R = 0.32; *p <* 1.9e^-13^) (Figure 10B), CD44 (R = 0.21; *p =* 1.4e^-06^) (Figure 10C), and SOX2 (R = 0.29; *p =* 2.2e^-11^) (Figure 10D). Drug sensitivity analysis using the GDSC database revealed distinct responses between the high- and low-DRS groups. The low-DRS group exhibited higher sensitivity to axitinib, imatinib, pazopanib, and cisplatin, whereas the high-DRS group displayed sensitivity to temsirolimus and gefitinib (Figure 10E). Furthermore, the potential predictive role of DRS in targeted therapy efficacy was explored in patients with ccRCC treated with everolimus in the RCC-Braun_2020 cohort [21]. Kaplan–Meier analysis showed that the low-DRS group had a significantly better overall progression-free survival (PFS) than the high-DRS group (log-rank test, *p <* 0.05; Figure 10F). Although not statistically significant, the KM curve for OS also suggested better clinical prognosis in the low-risk group (Figure 10G). Moreover, patients with ccRCC who responded to everolimus had significantly lower DRS scores than non-responders (Figure 10H).

### Renal cancer cells with high DRS display pronounced biological traits related to the malignancy

**Figure 11:**
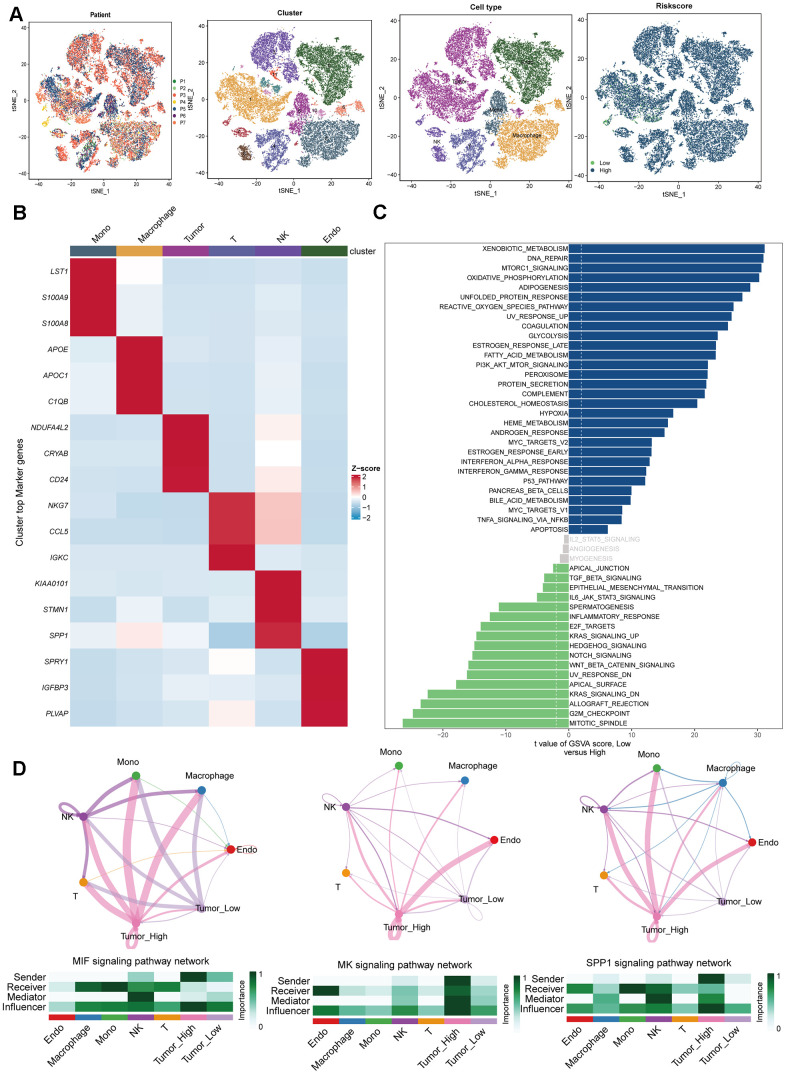
**Single-cell RNA-sequencing data analysis.** (**A**) Plots of 7 patients, 11 cell clusters, 6 cell types and different DRS level using t-SNE. (**B**) Marker gene heatmap of each cell subpopulation. (**C**) Gene set variation analysis of renal cancer cells with different levels of DRS. (**D**) Cellular interaction networks between renal cancer cells with different DRS levels and other cells in the MIF, MK, and SPP1 signaling pathways.

To validate the predictive utility of the DRS for ccRCC, additional analyses were performed using single-cell transcriptomic data from seven ccRCC samples (Figure 11A). This study aimed to determine whether DRS could delineate discrete biological features at the single-cell level. After quality control, normalization, integration, and principal component analysis (PCA), 11 clusters comprising 34,132 cells were identified (Figure 11B). Sub-cluster annotation revealed six cell types based on the cell marker website. DRS activity was quantified across B cells, endothelial cells, macrophages, monocytes, tumor cells, and T cells (Figure 11A, 11B) to assess the subtype-specific DRS profiles. Gene set variation analysis (GSVA) analysis of single-cell sequencing data revealed that tumor cells with high DRS exhibited increased activity of pathways associated with malignant phenotypes, including TGF-signaling, EMT, inflammatory response, KRAS signaling, Notch signaling, and Wnt/β-catenin signaling. In contrast, tumor cells with low DRS showed increased activity in metabolic pathways such as xenobiotic metabolism, fatty acid metabolism cholesterol homeostasis, heme metabolism, bile acid metabolism, oxidative phosphorylation, and glycolysis. Furthermore, they displayed elevated activity in immune pathways, including interferon alpha response, interferon gamma response, and complement signaling (Figure 11C). To investigate the communication patterns between tumor cells stratified by DRS status and other cell types, we used CellChat to identify ligand-receptor pairs and molecular interactions. We observed a specific cellular interaction between high-DRS tumor cells and immune cells within the ccRCC microenvironment. Specifically, in the MIF, MK, and SPP1 signaling pathways, high-DRS tumor cells acted as key senders and influencers communicating with immune cells (Figure 11D).

### DRS predicts the response of ccRCC to immunotherapy

**Figure 12:**
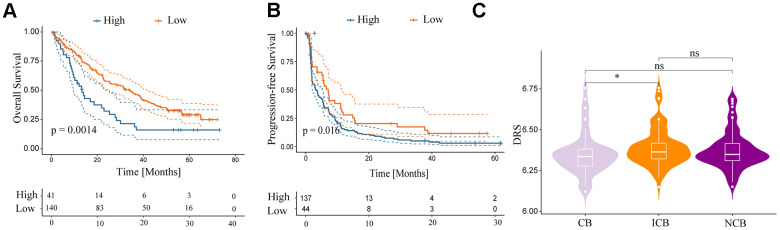
**Relationship between DRS and immunotherapy.** (**A**, **B**) Survival analysis for overall survival and progression-free survival of the two DRS groups in patients with ccRCC who were treated with nivolumab from the RCC-Braun_2020 cohort. (**C**) Comparison of DRS of different patients with ccRCC in different remission states after immunotherapy in RCC-Braun_2020 cohort. (ns, *p >* 0.05; *p < 0.05).

This study investigated the predictive value of the DRS in anti-PD-1 therapy among patients with ccRCC treated with nivolumab from the RCC-Braun_2020 cohort. Patients were categorized into high- and low-DRS groups. The low-DRS group showed a more favorable prognosis after PD-L1 treatment (*p <* 0.05; Figure 12A, 12B) and was more likely to benefit from anti-PD-L1 immune checkpoint treatment (Wilcoxon test, *p <* 0.05; Figure 12C). The low-DRS group showed a more favorable prognosis after PD-L1 treatment (*p <* 0.05; Figure 12A, 12B) and was more likely to benefit from anti-PD-L1 immune checkpoint treatment (Wilcoxon test, *p <* 0.05; Figure 12C). In conclusion, these findings suggest a possible association between a low DRS and positive therapeutic outcomes in immunotherapy.

### Effects of SLC7A11 on proliferation, migration, and invasion of ccRCC cells

**Figure 13:**
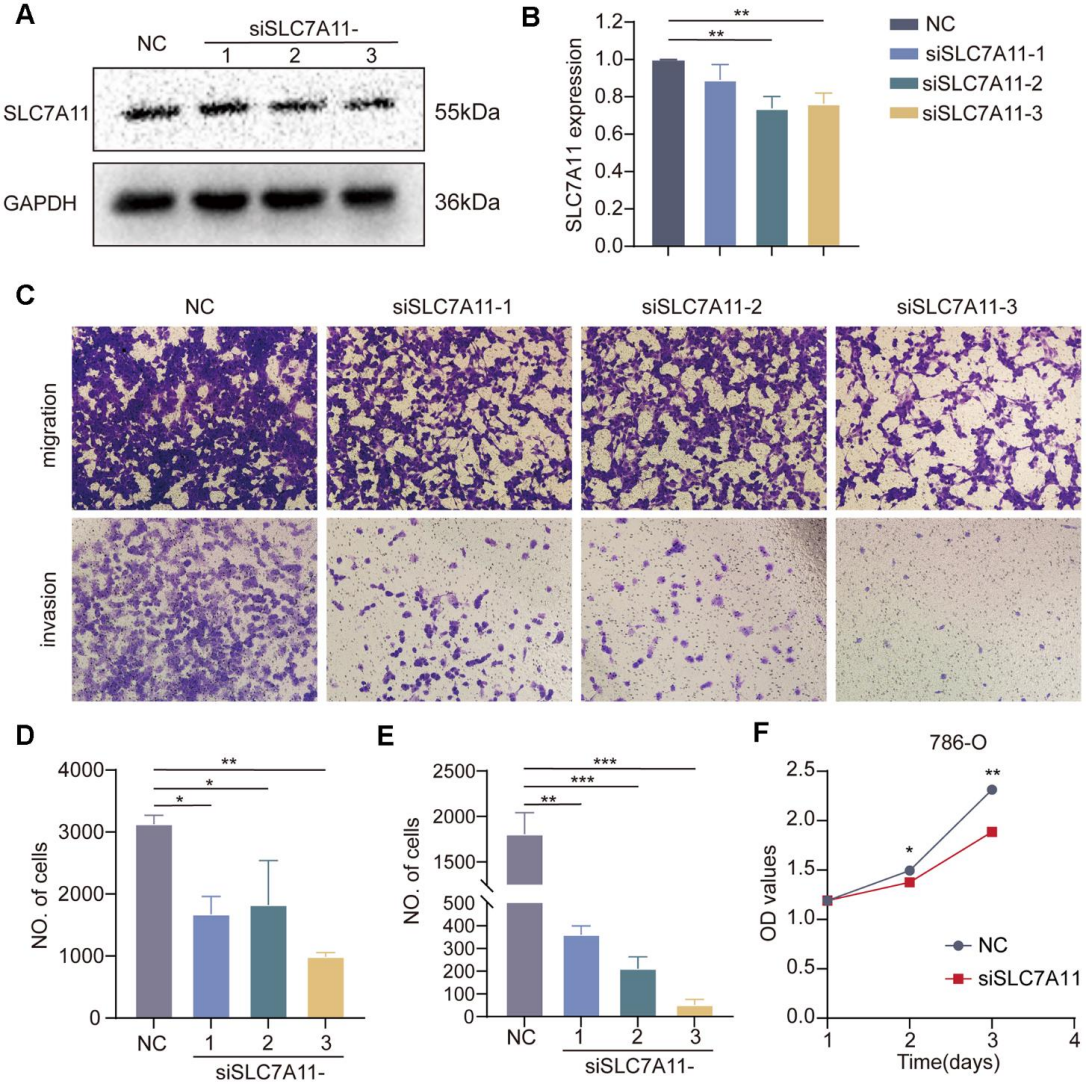
**Effect of SLC7A11 knockdown on the migration, invasion, and proliferation of renal cancer cells.** (**A**, **B**) Western blotting. (**C**) Transwell assay was performed to determine cell migration (**D**) and invasion (**E**). (**F**) Crystal violet assay to detect the proliferative capacity of renal cancer cells.

Solute Carrier Family 7 member 11 (SLC7A11) is a crucial gene involved in disulfidoptosis and plays a key role in tumor development and progression [22–24]. However, the precise role of SLC7A11 in the development of cancer remains unclear. Differential expression analysis conducted on the TCGA-KIRC dataset revealed that SLC7A11 was significantly upregulated in tumor tissues compared to that in normal tissues (Supplementary Figure 5A). DEGs analysis was performed on the TCGA-KIRC cohort using the ‘DESeq2’ package. The results indicated that 1032 DEGs were differentially expressed between the high and low SLC7A11 groups of ccRCC based on the criteria of *p <* 0.05 and |log2FC|>1 (Supplementary Figure 5B). The biological role of SLC7A11 in ccRCC was elucidated by GSEA. Functional HALLMARK, KEGG, and GO terms associated with SLC7A11 were analyzed. The enriched pathways are shown in Supplementary Figure 5C–5E. These results indicate that high expression of SLC7A11 is enriched in genes from proliferation- and metastasis-related pathways, including the E2F and EMT signaling pathways. Subsequently, *in vitro* experiments were performed to gain deeper insights into the impact of SLC7A11 on kidney cancer cell function. The three vectors for SLC7A11 siRNA knockdown were transfected into 786-O cells. Transfection efficiency was verified by western blotting, and the results are presented in Figure 13A, 13B, which show excellent transfection efficiency. Next, a Transwell chamber experiment was performed to verify the migration and invasion abilities (Figure 13). The migration, invasion, and proliferation abilities of 786-O cells were significantly reduced after SLC7A11 knockdown. The proliferation assay (Figure 13F) confirmed that SLC7A11 effectively affects the proliferation of ccRCC cells, showing that knockdown of SLC7A11 markedly reduces the proliferation ability of ccRCC cells in 786-O compared to that of negative cells. These findings suggest that SLC7A11 significantly affects the migration, invasion, and proliferation of renal cancer cells.

## DISCUSSION

The current approach for classifying ccRCC relies primarily on its histopathological features. With the advancement of large-cohort cancer projects, including TCGA, CPTAC, and Gene Expression Omnibus (GEO), and the progress of bioinformatics algorithms, researchers can now access genomic heterogeneity. Previous studies have identified ccRCC subtypes through genomic profiling [25–28], which advances the potential for precise, personalized therapeutic interventions in the management of ccRCC. Disulfidptosis is a mechanism of cell death that is distinct from apoptosis, ferroptosis, pyroptosis, necroptosis, and cuprotosis. It is characterized by abnormal disulfide cross-linking of actin cytoskeletal proteins [12]. Mechanistically, glucose starvation leads to an insufficient supply of NADPH, which blocks cellular reduction of cysteine to cystine. Consequently, numerous disulfide bonds are formed between the actin molecules, inducing disulfide stress. The Rac/WAVE regulatory complex (WRC)-actin-related protein 2/3 (Arp2/3) signaling pathway is activated by disulfide stress, which ultimately induces cell death. Furthermore, studies have shown that the polymerization of disulfide bonds within mitochondria can affect tumor progression [29]. Although Gan et al. identified several disulfidation-related genes, their expression characteristics and function in ccRCC remain unclear. Thus, we identified the main characteristics of disulfidoptosis in ccRCC, which can serve as a useful reference for guiding cancer treatment.

This study presents a comprehensive analysis of disulfidoptosis-related genes using multiomics data from over 10,000 samples from 33 cancer types. These findings indicate a significant downregulation of disulfidoptosis genes in cancerous tissues compared to that in normal tissues. This downregulation is associated with hypermethylation events and copy number variations. Based on disulfidoptosis signatures, ccRCC can be classified into two subtypes: DC1 (disulfidoptosis-cluster 1) and DC2 (disulfidoptosis-cluster 2). The DC2 subtype, characterized by its disulfidoptosis-desert phenotype, has a poor prognosis, with a higher TMB and MATH score, as well as reduced immune infiltration. The activation of disulfidoptosis has the potential to restructure tumor immunity within the ccRCC microenvironment by promoting antigen presentation.

To accurately evaluate the disulfidoptosis pattern of individual patients, we applied a methodology known as the disulfidoptosis-related score (DRS), which considers the individual heterogeneity of disulfidoptosis status. Integrated analyses showed that the DRS was a strong and independent prognostic factor for ccRCC.

To validate the robustness of DRS, we compared its performance with that of five previously published gene signatures based on programmed cell death-related genes. Comparative assessments demonstrated that the DRS had a superior prognostic ability compared to the other evaluated models. Although our model outperformed the five ccRCC models mentioned above, it may be too complex for clinical application. Therefore, future studies should focus on developing simplified gene signatures that maintain predictive accuracy while containing fewer genes. These streamlined models would balance precision and practicality, making them more suitable for widespread adoption in clinical settings. We related the DRS to the MoS classification established by Meng et al. and found that patients in the high-DRS group were more frequently classified as MoS1, whereas those in the low-DRS group were predominantly classified as MoS2 and MoS3. The MoS1 classification is characterized by a higher tumor stage, elevated hypoxia scores, and a higher frequency of SETD2 mutations, which are associated with poor prognosis. This finding helps explain the molecular characteristics that contribute to the poor prognosis of patients in the high-DRS group. In contrast, the MoS2 classification is associated with silent hypoxic signaling and a lower frequency of SETD2 mutations, which explains the favorable prognosis observed in patients with low DRS. Moreover, patients with MoS3 exhibit an activated tumor microenvironment, making them more responsive to immunotherapy. This is consistent with our findings.

It is widely recognized that clear cell RCC (ccRCC) lacks typical diagnostic markers. In this study, the diagnostic risk score (DRS) outperformed age in discriminating patients with ccRCC from normal samples, distinguishing metastatic patients from those with primary tumors, and identifying patients with advanced-stage ccRCC from those with early stage ccRCC, with higher sensitivity and specificity. Therefore, the DRS developed in this study has the potential to serve as a prospective biomarker for the diagnosis and prognosis of patients with ccRCC.

To improve clinical translatability, we integrated DRS with other clinicopathological parameters, such as age and the M stage, to create a user-friendly prognostic nomogram model. The results demonstrated that combining DRS with traditional clinicopathological factors can enhance prognostic accuracy, achieving a concordance index of 0.7795. This nomogram provides several advantages in clinical practice. Compared with single clinicopathological parameters, this model provides a more comprehensive and accurate assessment of patient risk and prognosis by integrating multiple prognosis-related factors. For instance, although staging pathological has long been a key prognostic determinant of renal cancer, sometimes relying solely on this parameter fails to effectively stratify high- and low-risk patients at the same stage. Our nomogram allows for a more precise prognostic prediction and individualized treatment strategies by enabling further risk stratification of patients within the same pathological stage. Furthermore, the nomogram displayed multiple prognostic factors, making complex risk prediction models easy to understand. Doctors should mark the patient’s condition on the corresponding line for each factor and then add the sum on the ‘total score’ line at the bottom to obtain the patient’s risk score and survival prediction. This nomogram has the potential to become a simple and efficient clinical decision support tool to guide individualized treatment strategies and follow-up management of renal cancer.

This study found that the DRS we created not only predicted the OS of patients with ccRCC but also showed significant positive correlations with TIC or CSC markers [19]. Increasing evidence supports the critical role of the TIC/CSC subpopulation in tumor initiation, progression, metastasis, and recurrence, which is attributed to properties such as self-renewal, multidrug resistance, and immune evasion [30–32]. The challenges in achieving complete tumor eradication are often attributed to these factors. Our study found a strong association between the DRS and tumor stemness. Furthermore, the group with a high DRS showed increased sensitivity to mTOR inhibitors, such as temsirolimus, but lower sensitivity to conventional chemotherapeutic agents, such as cisplatin. This suggests that tumors with high DRS may respond better to mTOR pathway inhibition. Finally, we validated the clinical utility of DRS in guiding adjuvant drug selection in the RCC-Braun_2020 cohort. Future prospective clinical studies are necessary to validate the application of the DRS model in therapeutic decision making.

This study used the GSE156632 single-cell RNA-sequencing cohort, which includes seven samples of clear cell RCC (ccRCC), to examine heterogeneity within the tumor microenvironment. Quality control and annotation using established marker genes identified six primary cell types: endothelial cells, tumor cells, T cells, natural killer cells, macrophages, and monocytes. GSVA of the single-cell data showed that tumor cells with a high Disulfidptosis-Related Score (DRS) had increased activity in pathways associated with malignant phenotypes, such as TGF Beta signaling, EMT, inflammatory response, KRAS signaling, Notch signaling, and Wnt/β-catenin signaling. In contrast, tumor cells with low DRS displayed elevated activity in metabolic pathways, such as xenobiotic metabolism, fatty acid metabolism, cholesterol homeostasis, heme metabolism, bile acid metabolism, oxidative phosphorylation, glycolysis, and peroxisomes, as well as immune pathways, including interferon alpha response, interferon gamma response, and complement. Furthermore, cell interaction profiling revealed significant communication between high-DRS tumor cells and infiltrating immune cell populations. This immune-tumor crosstalk promotes disease progression and dissemination, as infiltrating immune cells and soluble factors, such as cytokines, growth factors, chemokines, and exosomes, interact to establish an immunosuppressive microenvironment, allowing for the evasion of antitumor immunity. These findings collectively explain the poor clinical outcomes in the high-DRS subgroup.

Checkpoint inhibition has revolutionized the treatment of multiple advanced cancers, including ccRCC [33, 34]. The combination of immune blockade and targeted therapy has become the standard treatment for advanced patients with ccRCC [35]. However, there are still significant challenges to overcome, such as the limited number of patients who benefit from checkpoint blockade and the occurrence of severe adverse reactions associated with such therapies [36, 37].

Therefore, it is imperative to identify new therapeutic targets or adjuvants to assist immune therapy for ccRCC. According to the current paradigm in solid tumor immunology, the response to anti-PD-1 therapy is influenced by the pre-existing infiltration of CD8+ T cells and a high number of non-synonymous mutations [38–42]. However, unlike other types of cancer, the response to anti-PD-1 therapy in advanced ccRCC is not correlated with neoantigen load, TMB, or HLA zygosity [21]. Furthermore, there were no statistically significant differences in response to or survival following anti-PD-1 therapy between immune-infiltrated tumors and immune deserts/excluded tumors in advanced ccRCC [21]. This study verified the prognostic value of the DRS in anti-PD-1 therapy for patients with advanced ccRCC. The DRS has the potential to serve as a predictive strategy for anti-PD-1 therapy.

This study presents a novel perspective on immunooncology and individualized immunotherapy for ccRCC. However, it is important to acknowledge the inherent limitations of this study.

Bioinformatic analysis requires further experimental validation. The lack of clinical cohorts to verify the correlation between disulfidoptosis and the tumor immune landscape and the prognostic value of the DRS in ccRCC limited our analyses. Therefore, further validation based on a large-cohort prospective clinical trial is warranted. Based on a review of previous studies, we highlighted the role of SLC7A11 in ccRCC. Our results indicate that SLC7A11 is positively associated with immune response and EMT, suggesting its essential role in ccRCC metastasis and immunity.

## CONCLUSIONS

We present the initial systematic analysis of disulfidoptosis in clear cell RCC (ccRCC). The activation of disulfidoptosis may serve as a potential treatment approach for multiple cancers, and it plays a crucial role in tumor microenvironment remodeling. These findings enhance our understanding of tumor microenvironment cell infiltration and patient response to immunotherapy, which can promote personalized cancer immunotherapy in the future. SLC7A11 has been identified as a critical regulator of ccRCC progression.

## MATERIALS AND METHODS

### Data collection and processing

Supplementary Figure 1 presents the workflow of this study. The UCSC XENA dataset (http://xena.ucsc.edu/) [43] was used to download pan-cancer normalized expression profiling data, DNA methylation data, TMB, CNV, somatic mutation data, and clinical characteristics. The Express-Array database (https://www.ebi.ac.uk/arrayexpress/) was used to download an external ccRCC cohort, E-MTAB-1980, which includes expression profiles and prognostic information. To enhance comparability among datasets, we converted all RNA-seq data into transcripts per million (TPM) format. Furthermore, we obtained single-cell transcriptome data GSE121636 from the GEO database for further analysis. Because these datasets are publicly available and do not contain individual patient identifiers, ethical review committee approval and informed consent were unnecessary. The study excluded patients without prognostic information or expression profiles, as well as those who died within 30 days.

### Identification of distinct disulfidptosis subgroups in ccRCC

In a previous study, we identified genes associated with disulfidoptosis (DAGs), including SLC7A11, FLNA, FLNB, MYH9, TLN1, ACTB, MYL6, MYH10, CAPZB, DSTN, IQGAP1, ACTN4, PDLIM1, CD2AP, and INF2. We performed consensus clustering using the expression matrix of DAGs via the R package ‘ConsensusClusterPlus’ [44] and determined that the best classification number was k=2.

### Enrichment analysis between subgroups

Differential gene expression analysis was conducted between subgroups using the ‘DESeq2’ R package with count data. DEGs were identified using a p-value < 0.05 and an absolute log-fold change > 2 as thresholds. GSEA was then performed using the ‘clusterProfiler’ R package to elucidate the biological functions and molecular mechanisms underlying the differences between DC1 and DC2. |NES|> 1, NOM p-value < 0.05, and FDR q-value < 0.25 were established as the cut-off values. The gmt files necessary for enrichment analysis were obtained from the MSigDB database (https://www.gsea-msigdb.org/gsea/index.jsp) [45].

### Differences in immune infiltration signatures

Stromal and immune scores based on transcriptome profiling were evaluated using the R package ‘ESTIMATE.’ In each sample, the levels of 23 tumor-infiltrating immune cell types were quantified using single-sample GSEA (ssGSEA). The following cell types were included: activated B cells, activated CD4+ T cells, activated CD8+ T cells, activated dendritic cells, CD56bright natural killer cells, CD56dim natural killer cells, eosinophils, gamma delta T cells, immature B cells, and immature dendritic cells. MDSCs, macrophages, mast cells, monocytes, natural killer T cells, natural killer cells, neutrophils, plasmacytoid dendritic cells, regulatory T cells, T follicular helper cells, type 1 T helper cells, type 17 T helper cells, and type 2 T helper cells are all involved in the anticancer immune response. This response involves seven steps: (1) Cancer antigen release, (2) cancer antigen presentation, (3) activation, (4) immune cell transport to the tumor, (5) immune cell infiltration of the tumor, (6) recognition of T cells by cancer cells, and (7) destruction of cancer cells are the necessary steps for successful tumor remediation [46]. The gene data used in this analysis were obtained from the Tracking Tumor Immune Phenotype (TIP) website (http://biocc.hrbmu.edu.cn/TIP/index.jsp) and quantified using the single-sample GSEA (ssGSEA) algorithm to measure the levels associated with each step.

### Correlation between disulfidptosis and other related biological processes

To investigate the correlation between disulfidptosis and biological pathways, we used a collection of gene sets curated by Mariathasan et al. These gene sets include various biological processes, such as angiogenesis signature, antigen processing machinery, CD8 T-effector signature, cell cycle signature, cell cycle regulators, DNA damage repair, DNA replication, and EMT markers, such as EMT1, EMT2 and EMT3, Fanconi anemia signature, homologous recombination, immune checkpoint, mismatch repair, nucleotide excision repair, pan-fibroblast TGFb response signature (Pan-F-TBRS), and Wnt targets [47–49].

### Construction of a risk prediction model related to disulfidptosis

To create a prognostic model using these DEGs, several statistical analyses were conducted. Initially, we performed univariate Cox regression analysis to evaluate the prognostic significance of each DEG. We selected DEGs that were significantly associated with prognosis (*p <* 0.01) for further analysis. We then conducted a multivariate Cox regression analysis to identify independent prognostic biomarkers (*p <* 0.01). To reduce the risk of overfitting, we used LASSO regression analysis, which enabled us to determine the coefficient (β) for each gene included in the prognostic model. We then calculated the risk score for each patient in the TCGA database using the following formula: risk score = ∑ (βi * Ei), where βi represents the risk coefficient and Ei denotes the expression of each gene. Subsequently, we performed Kaplan–Meier survival curves combined with log-rank tests using the ‘survival’ and ‘survminer’ packages to assess the differences in survival between the low- and high-risk groups. ROC curves were generated to evaluate the sensitivity and specificity of the prognostic model in predicting the 1-year, 3-year, 5-year, and 10-year survival probabilities. The AUCs were defined using the “timeROC” package. Data from the E-MTAB-1980 cohort were used to validate the risk scoring model. Similar to the TCGA dataset, patients in both datasets were categorized into high- and low-risk groups based on their median risk scores. Furthermore, we performed the DeLong test to compare the predictive performance of the DRS, as quantified by the AUC, with that of other cell death-related gene signatures [50–55]. Statistical significance was set at *p <* 0.05. Univariate and multivariate Cox proportional hazard models were used to evaluate the prognostic ability of the DRS model, independent of other clinicopathological features, such as age, sex, histologic grade, pathologic stage, T stage, N stage, and M stage. The results were used to construct a prognostic nomogram by integrating the independent risk variables based on multivariate models. Calibration plots were used to measure the agreement between the predicted and observed survival events of the nomograms.

### Single-cell RNA-seq analysis

The GSE156632 dataset [56] was used to obtain single-cell RNA-sequencing data for seven ccRCC samples. The Seurat package [57] was used for cell clustering, dimensionality reduction, and other analyses. PCA was performed using RunPCA, followed by k-nearest neighbor analysis using FindNeighbors. RunTSNE was used for dimensionality reduction for visualization purposes. Cell type annotation was performed using known marker genes. Pseudotime analysis was conducted using the Monocle R package, and cell-cell interactions were analyzed with the CellChat R package.

### Evaluation of drug sensitivity and immunotherapy analysis

The ‘pRRophetic’ R package is a valuable tool for predicting clinical drug response using baseline tumor gene expression data. It utilizes statistical models constructed based on gene expression and drug sensitivity data from cell lines in the Cancer Genome Project (CGP). The ‘pRRophetic’ package in R software was used in our study to calculate the half-maximal inhibitory concentration (IC_50_) values of various chemotherapeutic and targeted agents for each patient in both the high-risk and low-risk groups using the TCGA-KIRC datasets. Furthermore, data were collected from the RCC-Braun_2020 cohort comprising 130 patients with ccRCC who received everolimus (32 patients with clinical benefit, 63 patients with intermediate clinical benefit, and 35 patients with no clinical benefit) and 181 patients with ccRCC who received nivolumab (57 patients with clinical benefit, 57 patients with intermediate clinical benefit, and 67 patients with no clinical benefit). The purpose of this study was to investigate the predictive ability of the DRS for targeted therapy and immunotherapy [21].

### Cell culture

Human 786-O cells obtained from the American Type Culture Collection (Manassas, VA, USA) were cultured in a CO_2_ incubator at 37° C. A specific small interfering RNA (siRNA) targeting the human gene SLC7A11 sequence was used. The siRNA sequence was 5′ UCAGAAACACCUGUGUAUGCA 3′ and was purchased from Fenghuishengwu company located in Hu Nan, China. For the transfection experiment, 786-O cells were seeded into six-well plates at a density of 4 × 10^5^ cells per well and incubated at 37° C in a 5% CO_2_ environment until complete adherence to the culture surface was achieved. The cells were then transfected using the Lipo3000 transfection reagent.

### Western blotting

The cells were treated with cell lysis buffer at 100° C for 10 min to extract total protein. Proteins were then loaded onto a 10% SDS-PAGE gel and electrophoresed at a constant voltage of 120V. Finally, the proteins were transferred to a PVDF membranes. The PVDF membrane was sealed with 5% skim milk powder for 1 h and incubated overnight at 4° C with primary antibodies. This was followed by a 1-hour incubation with secondary antibodies. After incubation, the membranes were washed three times with phosphate-buffered saline (PBS) and visualized. The relative abundance of proteins was assessed using Image Lab analysis software and ImageJ software.

### Cell proliferation assay

Cell viability was evaluated using a crystal violet assay. 786-O cells transfected with siRNA-SLC7A11 were harvested at 90% confluency and seeded into 96-well culture plates at a density of 2 × 10^3^ cells/well, with five replicate wells for each experimental group. The plates were placed in a 37° C, 5% CO_2_ incubator and assessed 24, 48, and 72 h post-seeding using a crystal violet assay. For the assay, the culture medium was aspirated from each well and 50 μL of 0.5% crystal violet staining solution was added. The plates were then incubated at room temperature on a bench rocker at a frequency of 20 oscillations/min for 20 min. After staining, the plates were washed with PBS to remove excess staining solution. Subsequently, the samples were air-dried overnight at room temperature. To quantify stained cells, 200 μL of methanol was added to each well and incubated for 10 min at room temperature. The optical density of each well was measured at 570 nm (OD570) by using a microplate reader. This measurement provided an indication of cell viability.

### Transwell migration and invasion assay

A 24-well Transwell chamber (8 μm, Thermo Fisher Scientific, Waltham, MA, USA) was prepared by coating the inserts overnight at 4° C with or without 100 mL of matrix gel substrate provided by BD Biosciences (San Jose, CA, USA). Next, 100 μL of cell suspension containing 3 × 10^4^ cells/mL was added to the Transwell inserts with or without the matrix gel substrate. The culture medium (600 μL) containing 10% FBS was added to the lower chamber. The cells were incubated in Transwell chambers for 48 h. After incubation, cells were fixed with 4% paraformaldehyde at room temperature for 20 min. The cells were then stained with 0.5% crystal violet for 5 min. The stained cells were then counted to evaluate their presence and distribution, allowing for the assessment of cell migration or invasion through the Transwell chambers, with or without the matrix gel substrate, using cell fixation and crystal violet staining techniques.

### Statistical analysis

Statistical analyses were conducted using R software (version 4.2.1) and appropriate packages. Kaplan–Meier survival analysis was used to establish survival curves, and the log-rank test was performed to compare the statistical significance of survival rates between the different risk groups. Univariate and multivariate Cox regression models were constructed to examine the prognostic power of clinicopathological variables and risk scores. Chi-square or Fisher’s exact tests were used to determine the association between risk scores and clinical characteristics. A two-tailed *p <* 0.05 indicated statistical significance (*p < 0.05; **p < 0.01; ***p < 0.001; ****p < 0.0001).

### Data availability

The corresponding author can be contacted for free to receive any data or R codes used in this study. All authors reviewed and approved the final draft of the manuscript. Publicly accessible datasets were also examined. These are accessible through GEO (https://www.ncbi.nlm.nih.gov), The Cancer Genome Atlas (https://portal.gdc.cancer.gov/), and UCSC Xena (http://xena.ucsc.edu).

### Consent for publication

All authors consent to the publication of this study.

## Supplementary Material

Supplementary Figures

Supplementary Table 1
