# Supplementary material for: A new perspective: deciphering the aberrance and clinical implication of disulfidptosis signatures in clear cell renal cell carcinoma
Source: Aging (Albany NY). 2024 Jun 10;16(11):10033–62. doi: 10.18632/aging.205916 (PMC11210246; doi:10.18632/aging.205916)
Supplement: Supplementary Figures [file aging-16-205916-s001.pdf]

SUPPLEMENTARY FIGURES

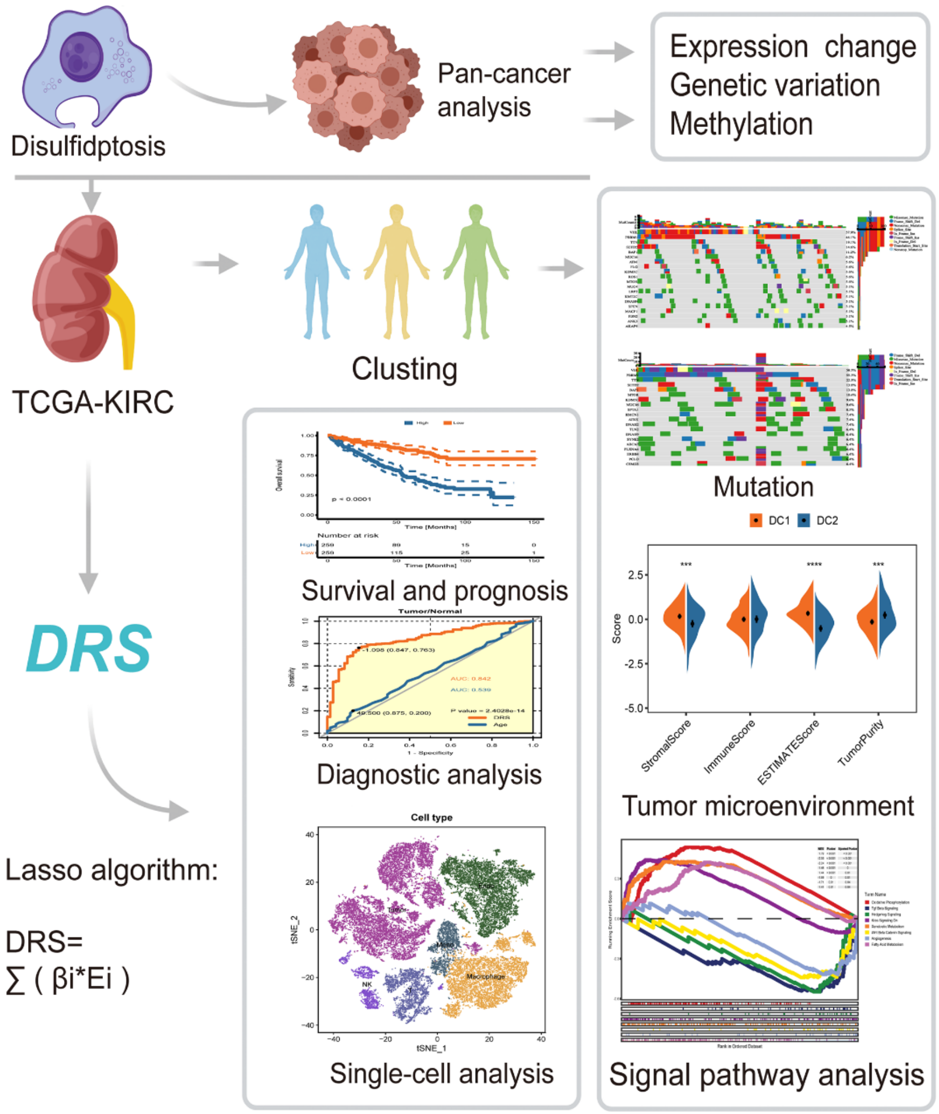

Supplementary Figure 1. Workflow of our research.

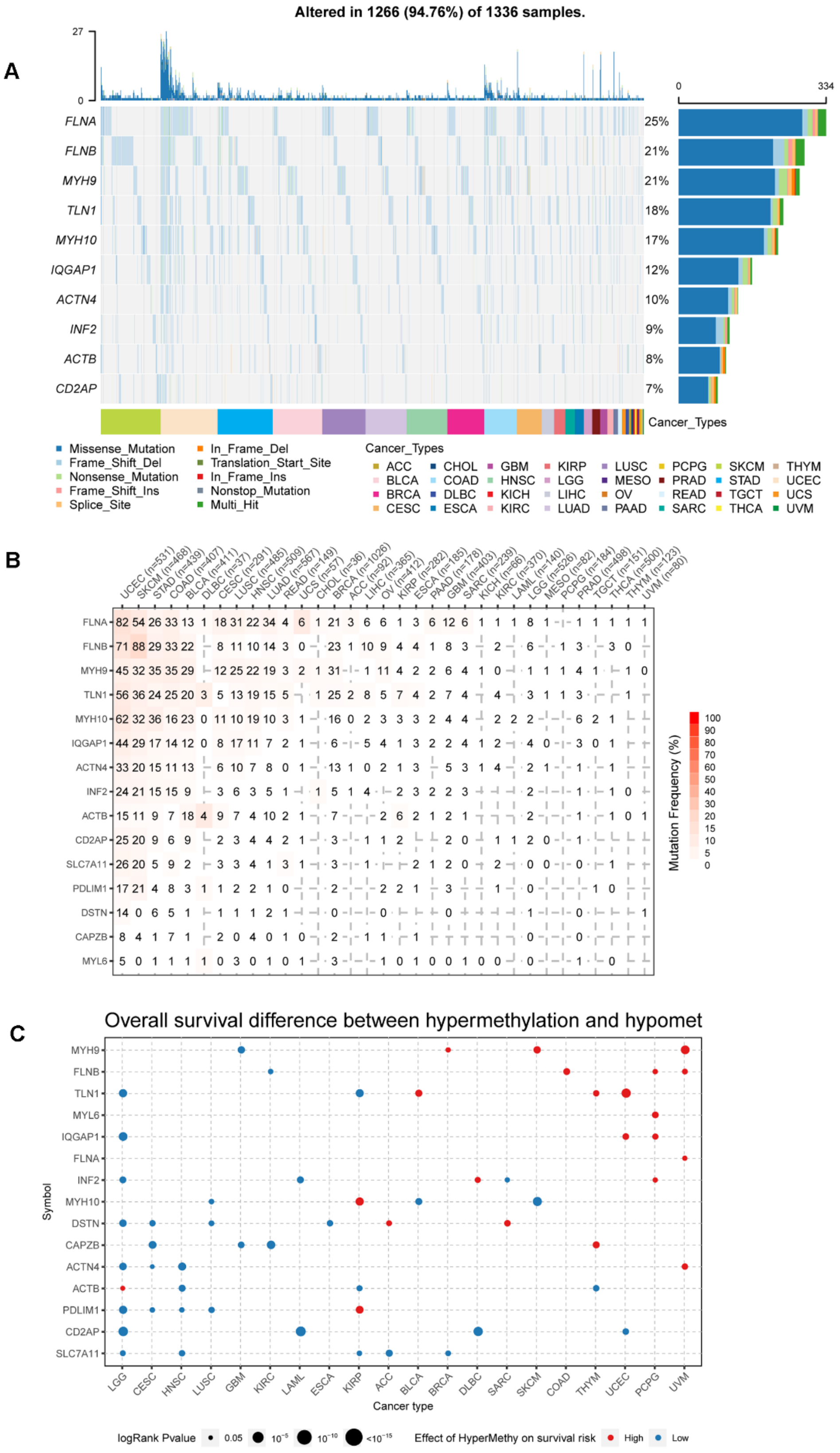

Supplementary Figure 2. Landscape of mutation and methylation of 15 disulfide-related molecules. (**A**) Waterfall diagram showing the mutation types of disulfidation-related genes in multiple cancers. (**B**) Heatmap showing the mutation frequency of 15 disulfide-related molecules in pan-cancer. (**C**) Bubble chart showing the correlation between methylation of 15 disulfide-related molecules and overall survival.

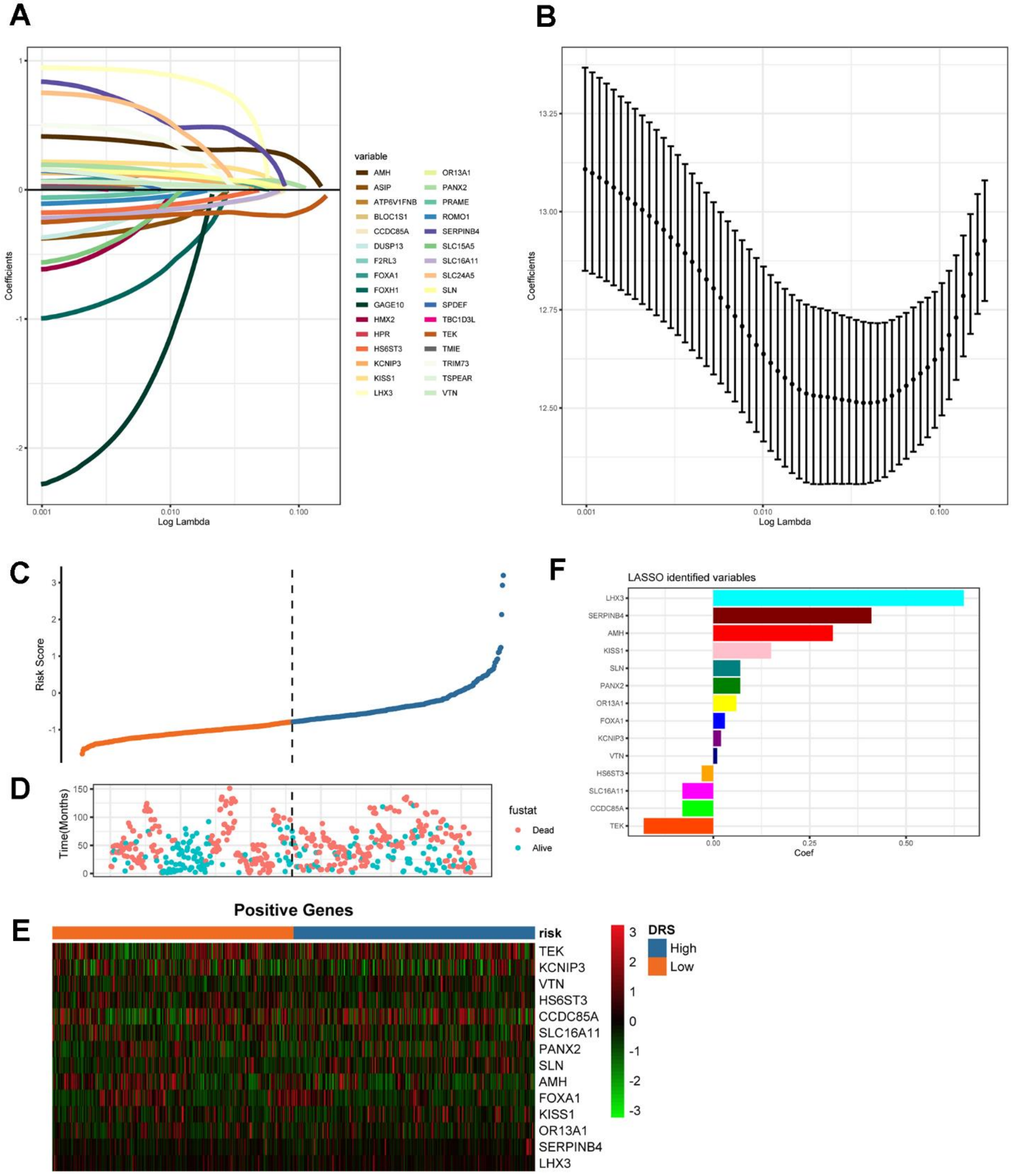

Supplementary Figure 3. Establishment of DRS by LASSO regression analysis. (**A**) LASSO coefficient profiles of the 32 DEGs in ccRCC. (**B**) Tenfold cross-validation LASSO regression analysis to estimate the optimal LASSO regularization parameter. (**C**, **D**) DRS distribution, survival status, and survival time of patients with ccRCC in the TCGA-KIRC dataset. (**E**) Heatmap of the mRNA expression of 14 DRS model-related genes in patients with ccRCC from TCGA-KIRC (n = 518) dataset. (**F**) Coefficients for 14 modeled genes.

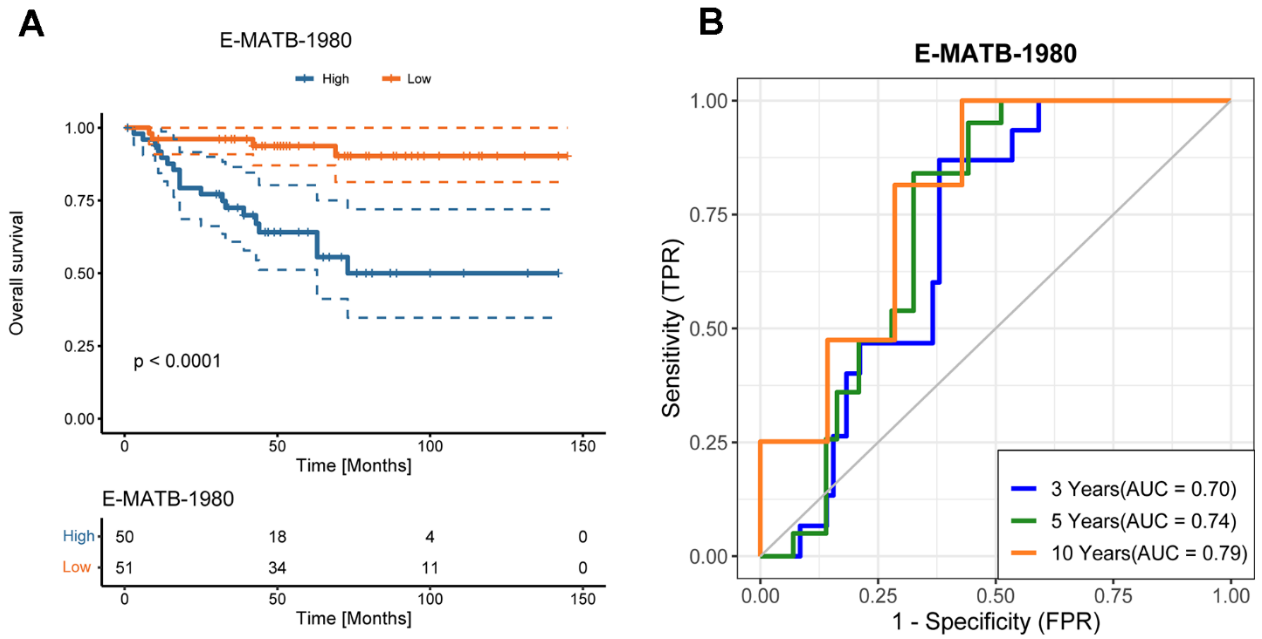

Supplementary Figure 4. Verify the prognostic value of DRS in the E-MATB-1980 cohort. (**A**) Survival analysis for overall survival of the two DRS groups in the E-MATB-1980 cohort. (**B**) Time-dependent ROC analysis of the predictive value of the DRS in the overall survival of patients at 1, 3, 5, and 10 years based on the E-MATB-1980 cohort.

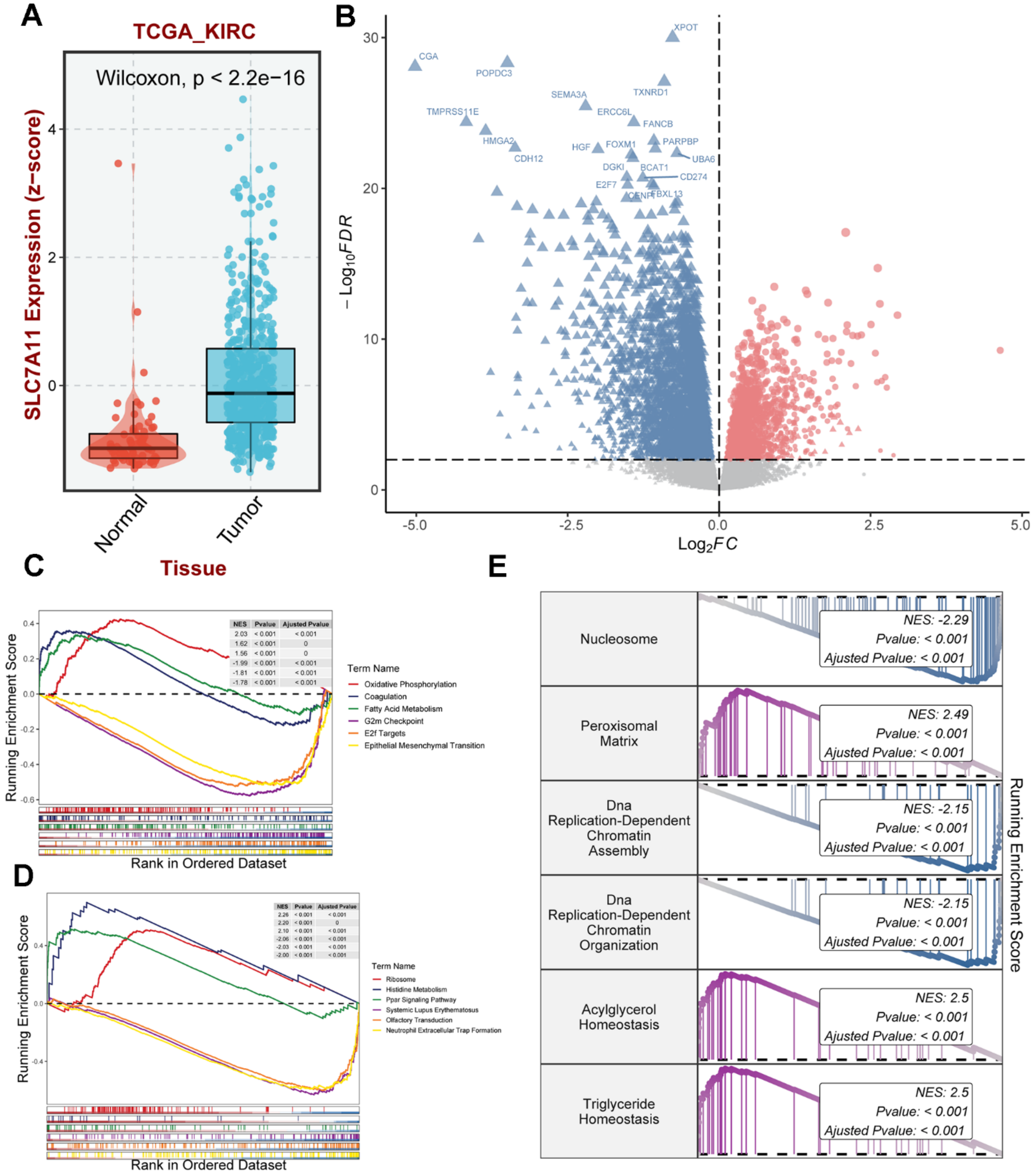

Supplementary Figure 5. Functional enrichment indicates that SLC7A11 is potentially associated with EMT and the TME in ccRCC. (**A**) The box plot compares the expression levels of the SLC7A11 gene between tumor and normal tissues. (**B**) Volcano plot of SLC7A11-related DEGs in ccRCC. Red and blue points indicate the upregulated and downregulated genes, respectively. (**C**) GSEA Hallmark analysis. (**D**) GSEA KEGG analysis. (**E**) GSEA GO analysis.
