# Supplementary material for: A new perspective: deciphering the aberrance and clinical implication of disulfidptosis signatures in clear cell renal cell carcinoma
Source: Aging (Albany NY). 2024 Jun 10;16(11):10033–62. doi: 10.18632/aging.205916 (PMC11210246; doi:10.18632/aging.205916)
Supplement: Supplementary Table 1 [file aging-16-205916-s002.pdf]

SUPPLEMENTARY TABLE

Supplementary Table 1. Variances in gene mutation frequencies between DC1 and DC2 groups.

Hugo_symbol	Group1	Group2	n_mutated_group1	n_mutated_group2	p_value
PBRM1	Cluster1	Cluster2	83 of 228	63 of 128	0.024531121
PBRM1	Cluster2	Cluster1	63 of 128	83 of 228	0.024531121
BAP1	Cluster1	Cluster2	21 of 228	16 of 128	0.367009091
BAP1	Cluster2	Cluster1	16 of 128	21 of 228	0.367009091
TTN	Cluster1	Cluster2	34 of 228	24 of 128	0.371288981
TTN	Cluster2	Cluster1	24 of 128	34 of 228	0.371288981
VHL	Cluster1	Cluster2	108 of 228	58 of 128	0.740531199
VHL	Cluster2	Cluster1	58 of 128	108 of 228	0.740531199
MUC16	Cluster1	Cluster2	17 of 228	9 of 128	1
MUC16	Cluster2	Cluster1	9 of 128	17 of 228	1
